# Biological analysis of *Sonchus oleraceus* (Linn) extract and its effect on mitigating sodium benzoate-induced cytotoxicity and genotoxicity

**DOI:** 10.3389/ftox.2025.1674822

**Published:** 2025-11-13

**Authors:** Maryam Abdulmalik Althubyani, Abdulmajeed F. Alrefaei, Sameer Hasan Qari, Rayan S. Alharbi, Aljawharah Alqathama

**Affiliations:** 1 Department of Biology, Jamoum University College, Umm Al-Qura University, Makkah, Saudi Arabia; 2 Department of Biology/Genetic and Molecular Biology Central Laboratory (GMCL), Jamoum University College, Umm Al-Qura University, Makkah, Saudi Arabia; 3 Biology Department, Jamoum University College, Umm Al-Qura University, Makkah, Saudi Arabia; 4 Biology Department, Faculty of Applied Science, Umm Al-Qura University, Makkah, Saudi Arabia; 5 Department of Pharmaceutical Sciences, Pharmacy College, Umm Al-Qura University, Makkah, Saudi Arabia

**Keywords:** *Sonchus oleraceus* (Linn), medicinal plant, genotoxicity, sodium benzoate, ISSR PCR, *Allium cepa*

## Abstract

**Background:**

Folk medicine has long employed plants to treat diseases. Consumers believe herbal treatments are safe since they are natural. Studies suggest that some plant compounds can cause chromosomal damage at high concentrations, while some can mitigate the genotoxicity caused by toxic substances. *Sonchus oleraceus* L. is a popular medicinal herb in Saudi Arabia as well as in the rest of the world. It has antioxidant, anticancer, and other biological properties. Sodium benzoate (SB) is a versatile food preservative used in packaged food and drink industries; it has been found to cause genotoxicity and DNA damage. Therefore, it is necessary to investigate the biological activity of *Sonchus oleraceus* extract and its ability to mitigate Sodium Benzoate-induced cytotoxicity and genotoxicity.

**Aim:**

The current study evaluates biological properties of *S. oleraceus* leaf extract and reveals its potential mitigating role against sodium benzoate by using the *Allium cepa in vivo* bioassay and molecular analysis.

**Methodology:**

*S. oleraceus* aqueous extract and sodium benzoate was prepared. Then, the effective concentration (EC_50_) was determined, and concentrations with control were selected for each group. Roots of *A. cepa* were treated for 24, 48, and 72 h with concentrations (21.5, 43, and 64.5 mg/mL) of extracts with or without combined treatment with 4 mg/mL of SB for 24 h. The cytotoxicity was investigated by using mitotic index (MI) and the genotoxicity by micronuclei (MN), chromosomal abnormalities (CA) and then using the ISSR-PCR markers for molecular analysis.

**Results:**

Compared to the controls, *S. oleraceus* and SB application as a single treatment decreased root length and MI index, and CA were increased, especially in higher concentrations. DNA damage was reported by ISSR-PCR markers. However, SB toxicity was mitigated by the co-treatment of *S. oleraceus* extract, which showed partial improvement in all variables depending on the application concentration, possibly due to its antioxidant properties. The cytogenetic assay showed the best antimutagenic efficacy at 21.5 mg/mL with a moderate inhibition rate greater than 25%.

**Conclusion:**

The results indicate that the aqueous extract of *S. oleraceus* leaves, as a single treatment, induces a genotoxic effect on *A. cepa* cells, especially at high concentrations, and that *S. oleraceus* leaf extract, as a co-treatment, acts as a mutagen at high concentrations and a moderate antimutagenic at low concentrations. The findings also indicate that the cytotoxic capacity of SB in *A. cepa* highlights potential concerns that warrant further investigation.

## Graphical Abstract

**Figure:**
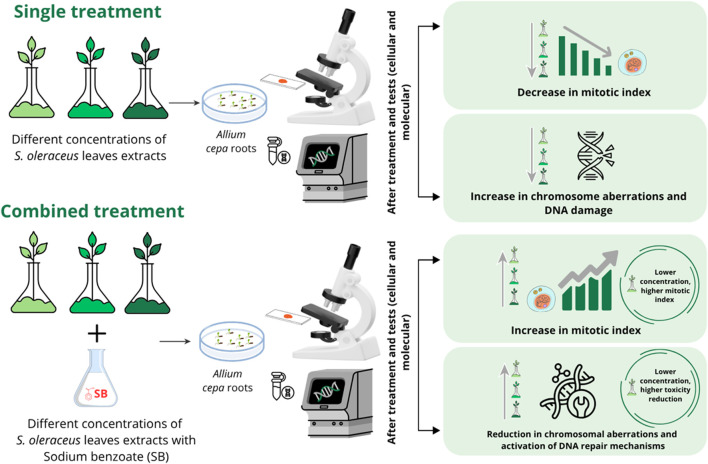

## Introduction

Medicinal plants represent a significant source of healthcare in the Arab East because they are essential components of prophetic medicine (Almutairi and De Santis, 2024). In Saudi Arabia, vast majority of the population uses medicinal plants in traditional medicine for illness prevention (Ali et al., 2024). According to the World Health Organization (WHO), approximately 80% of the developing nations world’s population relies on medicines extracted from medicinal plants to treat many diseases or for primary healthcare (Ekor, 2014). Saudi Arabia is rich in medicinal plants that have shown promising results for anti-cancer and antioxidant properties (Sher and Aldosari, 2012; Alzandi et al., 2021; Daradka et al., 2021). More than 10% of plant species (50,000 species) are used for beauty and therapeutic purposes (Verma and Singh, 2008). The active compounds are found in different parts of medicinal plants such as seeds, roots, leaves, fruits, or the complete plant, can provide direct or indirect therapeutic effects (Hilal et al., 2024; El-Saadony et al., 2025).

While medicinal plants are generally considered safe, many studies have revealed that medicinal herbs have negative side effects, including possible toxicity (Boukandou Mounanga et al., 2015). The toxicity of therapeutic plants depends on their chemical composition; even low-toxicity extracts may elicit harm with extended use. Several studies have revealed that plants commonly used in folk medicine could cause genotoxic effects (Marques et al., 2003; An et al., 2010; Melo-Reis et al., 2011; Regner et al., 2011; Shin et al., 2011; Sponchiado et al., 2016). For example, extracts from *Argyrolobium roseum* showed cytotoxic and genotoxic activities *in vitro*, which raised worries about possible harm to DNA (Rasool et al., 2023). In a similar vein, whole-plant extracts of *Kalanchoe laciniata* have shown both cytotoxicity and mutagenic potential in the Ames and MTT experiments (Sharif et al., 2017). The micronucleus assay was used to assess the hormetic response of *Cistus monspeliensis* leaf extract, which showed protective effects at low concentrations but possibly harmful genotoxic effects at higher dosages (Al-Naqeb et al., 2024). These results highlight how crucial it is to incorporate targeted toxicological analyses into research on therapeutic plants.

The use of food additives has increased enormously in the last few decades (Montera et al., 2021; Dunford et al., 2023). Sodium benzoate (SB) is one of the most widely used food additives; it is a sodium salt with a molecular weight of 144.1 g/mol, C_7_H_5_O_2_Na formula, odorless, and soluble in water and ethanol. It is used in cosmetics, pharmaceutical, and food industries as a preservative (Lennerz et al., 2015), and in food and drinks due to its ease of application and efficacy in inhibiting the proliferation of fungi and bacteria during storage (Tsay et al., 2007). Studies have shown that sodium benzoate consumption leads to several harmful effects, including genotoxicity and DNA damage (Aledwany et al., 2018; Acar, 2021; Ali et al., 2020). Moreover, it can cause liver and renal impairment (Afshar et al., 2012). It leads to reduction in the mitotic index and increased sister chromatid exchanges (SCEs), chromosomal aberrations (CA), and micronuclei (MN) of human lymphocyte cells (Yılmaz et al., 2009). Also, it induces oxidative damage by reduced glutathione levels in murine brain tissue and heightened MDA levels (Khoshnoud et al., 2018). It provokes oxidative stress in zebrafish (Gaur et al., 2018) and in *Allium cepa* cells (Acar, 2021). It causes an increase in MDA levels coupled with a decrease in SOD and CAT activities in human erythrocytes (Yetuk et al., 2014). Since multiple studies have shown SBs’ harmful effects, we aimed to explore ways to reduce these effects. Oxidative stress is a cellular state defined by an imbalance between the generation of reactive oxygen species (ROS) and the antioxidant defense systems. Excessive reactive oxygen species, including hydroxyl radicals, superoxide anions, and hydrogen peroxide, can result in lipid peroxidation, protein oxidation, and, most importantly, DNA damage through base alterations, single- and double-strand breaks, and chromosomal abnormalities (Chen G.-H. et al., 2022; Shields et al., 2021; Juan et al., 2021). Cells initiate intricate DNA damage response (DDR) pathways to mitigate these consequences, encompassing damage identification, cell cycle arrest, and DNA repair mechanisms, including base excision repair (BER) (Li et al., 2021).

Medicinal plants have not been studied extensively in the regard of their genetic toxicity or their abilities to lessen the toxicity of food additives. Some medicinal plants possess the capacity to mitigate the cytotoxicity and genotoxicity induced by food additives, especially preservatives (Althubyani et al., 2024). However, they may also increase toxicity due to crude extracts consisting of a complex mix of phytochemicals that may interact synergistically, additively, or antagonistically (Chen X. et al., 2022).

*Sonchus oleraceus* L. is a medicinal plant belonging to the Asteraceae family, common in Saudi Arabia, and used in folk medicine to cure conditions like ulcers, skin infections, wounds, and painful scorpion stings (Awadh Ali et al., 2017; El Ghazali et al., 2010; Tounekti et al., 2019). Also, they were used for treating gastrointestinal ailments (Hussain et al., 2010), liver disorders and as a febrifuge, sedative, and vermifuge. An ointment decoction used to treat wounds and ulcers (Khare, 2007). *S. oleraceus* is used as antidiabetic (Teugwa et al., 2013) anti-inflammatory, antipyretic (Couto et al., 2011), antinociceptive, anxiolytic (Couto et al., 2011), and antibacterial (Elkhayat, 2009; Guo et al., 2011).

*S. oleraceus* is recognized for its abundance of phenolic chemicals and flavonoids, which have significant antioxidant capabilities. Numerous studies have indicated that extracts from *S. oleraceus* possess significant radical scavenging capabilities, particularly against DPPH and hydroxyl radicals, which is attributed to their high total phenolic and flavonoid content (Yin et al., 2007; Sánchez-Aguirre et al., 2024). Furthermore, these extracts have demonstrated the ability to augment the activity of endogenous antioxidant enzymes, including superoxide dismutase (SOD) and glutathione peroxidase (GPx), diminish lipid peroxidation indicators such as malondialdehyde (MDA), and regulate inflammatory signaling pathways, including the NF-κB and TLR4 pathways (Chen et al., 2020).

Despite *S. oleraceus’* extensive history of therapeutic use, little is known about its safety, particularly regarding its genotoxic and antigenotoxic effects. Some studies have investigated its cytotoxic and antioxidant properties; however, the results present a multifaceted picture. El Gendy et al. (2024) indicated that the essential oil demonstrated notable antioxidant activity and cytotoxicity against HepG2 cancer cells, implying potential advantageous and detrimental effects contingent upon the context. Yin et al. (2007) likewise found that extracts show strong radical-scavenging and reducing activity, indicating a protective role against oxidative stress that could lead to antigenotoxic effects. A recent study by Abdelhameed et al. (2025) revealed that *S. oleraceus* provides hepatoprotective effects in rats by activating the Nrf2/KEAP1/HO-1 signaling pathway, leading to increased antioxidant enzyme production, reduced oxidative stress, and alleviation of paracetamol-induced liver damage.

Due to the increased use of *S. oleraceus* in folk medicine and sodium benzoate as a food preservative, and the scarcity of data on the cytotoxicity, genotoxicity, or potential of *S. oleraceus* to mitigate sodium benzoate toxicity in plants, further investigation is warranted. Additionally, *S. oleraceus* having a phytochemical profile rich in antioxidants, so it was chosen for the current study to investigate its possible mitigating effects against sodium benzoate-induced toxicity. Therefore, this study investigates the following question: What is the impact of *S. oleraceus* extract on the meristematic cells of *A. cepa*, and does it mitigate the toxicity caused by sodium benzoate? Accordingly, the primary aim of this study is to assess the cytotoxic and genotoxic effects of *S. oleraceus* leaf extract, and to determine its mitigating potential against sodium benzoate toxicity in *A. cepa* cells through cytogenetic and molecular tests.

## Materials and methods

### Collection of plant samples

The leaves of *S. oleraceus* (Supplementary Figure S1) used for this study were collected from the city of Taif in the western region of Saudi Arabia at latitude 21° 16′13.01″N and longitude 40° 24′56.99″E. The samples were collected in December 2022 and received authentication from a taxonomist at Umm Al-Qura University in Saudi Arabia.

### Preparation of aqueous extracts

**FIGURE 1:**
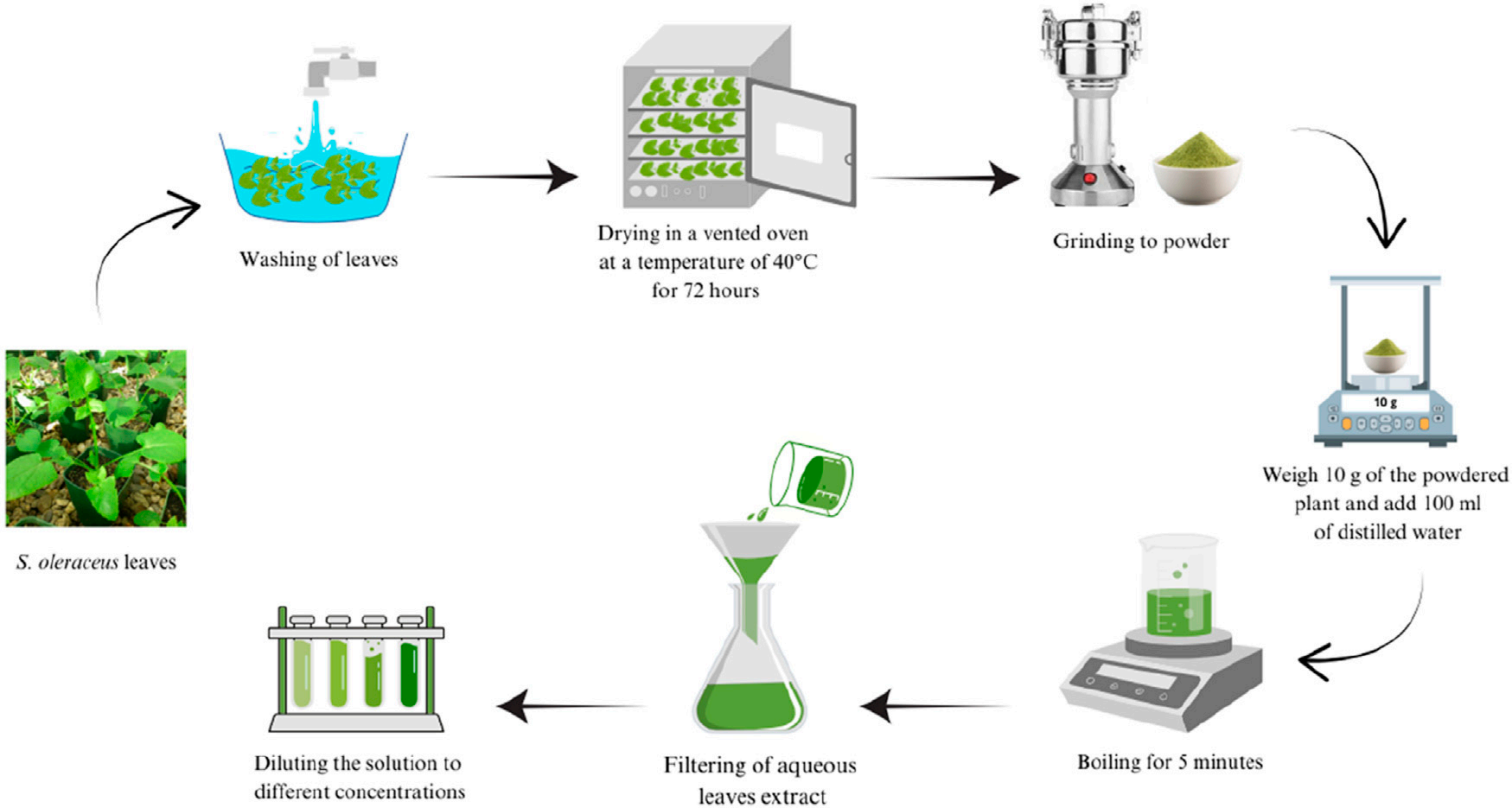
preparing an aqueous extract of *Sonchus oleraceus* leaves.

The crude extracts of *S. oleraceus* leaves were prepared according to the traditional use in Saudi Arabia as decoction (Figure 1). The aqueous extract of the dried leaves of *S. oleraceus* was prepared according to Çelik and Aslantürk (2010) with minor modifications (The temperature was reduced from 55 °C to 40 °C, and the drying time was increased from 24 h to 72 h). The leaves of *S. oleraceus* were harvested, washed several times using tap water, then washed with distilled water, and dried in a vented oven at 40 °C for 72 h. Then ground using an electric blender to obtain a fine powder. 10 mg of dry powdered leaves were boiled with 100 mL of dH_2_O (10% stock solution) in a covered beaker for 5 min. It was cooled at room temperature for 10 min and then filtered with Whatman filter paper No.1 to remove particulate matter. The extract was diluted with distilled water to prepare different concentrations, and the extract was used within 24 h.

### Preparation of sodium benzoate

To prepare Sodium benzoate, it was dissolved to a concentration of 4 mg/mL in distilled water. The Sodium benzoate used in these treatments was obtained from Sigma-Aldrich (USA), Catalogue Number (18106-1KG-R).

### Assessment of root growth inhibition and EC_50_

To calculate the EC_50_, 40 roots per initial concentration were examined for both the sodium benzoate (SB) and *S. oleraceus* treatments. A variety of concentrations of *S. oleraceus* extract (10, 20, 30, 40, and 50 mg/mL) and (1, 2, 3, 4, and 5 mg/mL) of Sodium benzoate were employed to determine the EC_50_ value and assess the inhibitory effect on root growth of *A. cepa* through the measurement of root length. *A. cepa* seeds (Emerald Seed Co., USA) were washed with dH_2_O, followed by the planting of 40 seeds in each Petri plate using sterile filter paper. The seeds were planted for 5 days using 4 mL of dH_2_O and thereafter subjected to 4 mL of each concentration once simultaneously. Certain seeds that were subjected to dH_2_O treatment were designated as the control group. The seeds germinated in darkness at 25 °C. The percentage inhibition of root growth in comparison to the control for each extract was assessed, subsequently identifying the effective concentration that reduced root growth by 50% relative to the control (Acar, 2021; Akwu et al., 2019; Qari, 2017).

In root growth experiments, 40 roots were analyzed for both single (*S. oleraceus* extract alone) and combined (SB + *S. oleraceus* extract) treatments per concentration and exposure duration. The EC_50_ values for *S. oleraceus* extract (43 mg/mL) and SB (4 mg/mL) were determined from root growth inhibition data by linear regression of the inhibition ratio against concentration (40 roots per concentration). Two additional concentrations of *S. oleraceus* extract were chosen based on this value: 1½ EC EC_50_ 50 (21.5 mg/mL) and 1.5 × EC_50_ (64.5 mg/mL). To verify the biological relevance and efficacy of these concentrations, root growth inhibition experiments were conducted at 24, 48, and 72 h utilizing the chosen concentrations.

### *Allium cepa* assay

Three slides per concentration and time point were used for cytogenetic analysis; these slides included 9 roots in total, and 3,000 cells were scored. To conduct cytogenetic and molecular tests for *S. oleraceus* extracts, *A. cepa* seeds were washed with dH_2_O. Then, forty seeds were sown per petri dish containing sterile filter papers. The seeds were germinated for 5 days using 4 mL of dH_2_O. After this period, they were subjected to separate treatments (single) using 4 mL of different concentrations of 21.5, 43, and 64.5 mg/mL of *S. oleraceus* extract. A portion of the seeds treated with dH_2_O served as the control. The combined treatments were performed as post-treatments as follows: the roots were exposed to a chosen concentration of Sodium benzoate (4 mg/mL) for 24 h, followed by a selected concentration of *S. oleraceus* [SB+SO] for 24, 48, and 72 h. Root tips treated with 4 mg/mL SB transferred to distilled water were used as a positive control. Despite SB operating by a different mechanism compared to standard mutagens, its proven genotoxicity makes it a suitable positive control for this study. Root tips treated with distilled water alone were used as a negative control (dH_2_O). The root tips measuring 2 cm from each seedling were collected and preserved in Carnoy’s fixative, composed of a 1:3 ratio of acetic acid to alcohol, for a duration of 24 h. It then proceeded to slide preparation or was stored in 70% alcohol (Sarhan, 2010).

### Slide preparation and examination

The procedure of Ping et al. (2012) was followed for the slide preparation with slight modifications. After pre-treatment, the washed root tips were placed into a fixative solution for 1 h and hydrolyzed in 1 N HCl at 60 °C–70 °C for 5 min. The root tips were washed five times with dH_2_O. Root tips 2 mL were cut and placed in a slide, and then three drops of acetocarmine stain were added and let to sit for approximately 2 min. The coverslip was carefully placed on the slide to prevent the formation of air bubbles and subsequently dried using tissues. The slides were examined using a ×10 objective lens to identify distinct clusters of cells, while a ×40 lens was employed to magnify the cells and observe chromosomes.

### Chromosomal aberrations and mitotic index analysis

The numbers of divided and non-divided treated cells of *A. cepa*, with the various phases of mitosis, chromosomal abnormalities, and mitotic abnormalities, were counted and photographed. The prevalent chromosomal abnormalities were observed, including bridges, chromosomal fragments, sticky chromosomes, and micronuclei. For each group and exposure duration, three slides containing nine *A. cepa* root tips were prepared, and 3,000 cells for mitotic index (MI) were counted in each slide.

The mitotic index was calculated according to Kusumaningrum *et al* (Kusumaningrum et al., 2012), as follows

$$\text{Mitotic index }\left(\text{MI}\right)=\frac{\text{Number}\;\text{of}\;\text{dividing}\;\text{cells}}{\text{Total}\;\text{number}\;\text{of}\;\text{cells}}\times 100$$

The percentage of total chromosomal aberration was calculated using the following formula:

$$\text{Chromosomal aberration }\%\;\left(\text{CA}\right)=\frac{\text{number}\;\text{of}\;\text{total}\;\text{aberrations}\;\left(\text{TA}\right)\;}{\text{total}\;\text{number}\;\text{of}\;\text{cells}\;\text{counted}\;\left(\text{TCC}\right)}\times 100$$

### DNA extraction

After 48 h of treatment, *A. cepa* roots were washed several times with distilled water for 10 min, then 200 mg of roots were pulverized in a microfuge for 2 min with 500 μL CTAB. The CTAB technique was employed to extract *A. cepa* DNA as outlined by Aboul-Maaty and Oraby and Fiona (Aboul-Maaty and Oraby, 2019; Clifford et al., 2022) and certain modifications were implemented. According to the Thermo Fisher Scientific nanodrop manual, the purity and concentration of DNA were measured for all samples using a nanodrop spectrophotometer. Agarose gel electrophoresis (2 g agarose was dissolved in 100 mL 1X TBE) was conducted to assess DNA quality visually. Then, each DNA sample was diluted to 25 ng/μL concentration, and samples were stored at −20 °C.

### ISSR-PCR markers

To evaluate the genotoxic potential of *S. oleraceus* leaf extracts or/and SB, ISSR-PCR markers were employed to detect polymorphism parentage. Initially, 10 ISSR primers were evaluated, and only four yielded distinct and informative banding patterns appropriate for analysis. Begin by extracting DNA from the *A. cepa* root, and then apply 4 different ISSR primers (ISSR418, ISSR-HB12, ISSR-UBS-811, and ISSR-MAO) was obtained from Macrogen, South Korea (Table 1). The annealing temperature for each primer was adjusted based on the manufacturer’s recommendations. ISSR PCR analysis was performed on a total of eight samples: three samples of *S. oleraceus* leaf aqueous extract, three samples representing different concentrations of combined treatment (SB + *S. oleraceus*), one negative control (dH_2_O) sample, and one positive control (SB) sample.

**TABLE 1:** ISSR Primers used in the present study.

Primer name	5′—3′ sequence	Melting timp °C
ISSR 418	CTCTCTCTCTCTCTCTTG	53
ISSR UBC-811	GAGAGAGAGAGAGAGAC	52.5
ISSR HB12	CAGCAGCAGGC	38
ISSR MAO	CTCCTCCTCCTCRC	46

A total volume of 25 μL was utilized for one polymerase chain reaction (PCR); this volume consisted of 10 μL 2x master mix, 2 μL 10 μM primer, 4 μL DNA template, and 4 μL nuclease-free water. Additionally, the master mix comprises compounds such as dNTP, MgCl_2_, Taq polymerase, and assay buffer. The PCR thermal cycle was started, repeated 35 cycles, left the PCR product at 72 °C for 7 min for the final extension, and held the last step at 4 °C (Eroz Poyraz et al., 2018; Poyraz, 2021). Then, gel was electrophoresed at a voltage of 100 V for a duration of 1 h and 20 min. Subsequently, the gel was introduced into a Gel Documentation system, where the bands were scrutinized and juxtaposed using ultraviolet (UV) light (Haglund, 2022). Following the PCR process, the gel electrophoresis bands were analyzed for all samples using Quantity One analysis software version 4.6.2. ISSR-PCR was conducted for all concentrations and treatments at a single time point (48 h). Although the assay was repeated multiple times to ensure clear band patterns in gel electrophoresis, the final analysis was based on single runs without triplicate data points.

### Data analysis

Statistical analysis was conducted using R software (R Core Team R, 2025) and SPSS Statistics version 29.0.1. Descriptive statistics (mean ± SD) were derived for each concentration by duration of exposure. To assess whether exposure to different concentrations of plant extraction *S. oleraceus* (SO) and their duration of exposure resulted in cytotoxic and genotoxic effects on onion’s root tips, we performed one-way ANOVA, modelling mitotic index, chromosomal aberration, and mutation index as response variables. The model included duration of exposure (24, 48, and 72 h) and concentrations (control = 0 mg/mL, 21.5 mg/mL, 43 mg/mL, and 64.5 mg/mL of SO) and interaction between them as a factor. Tukey adjusted *post hoc* pairwise comparisons were performed using emmeans package (Lenth et al., 2025) to detect differences within factor levels. A p-value ≤0.05 was considered statistically significant. In a subsequent analysis, the combined treatment concentrations were initially tested across all three exposure periods (24, 48, and 72 h). Because the effect of SO as a combined treatment follows the same trend between 48 h and 72 h and offers no more insights, I used 24 h and 48 h time points to test the effect of SO as a mitigating agent compared to positive control (SB) and negative control (dH_2_O). The comprehensive statistical analyses, encompassing the 72 h duration for the combination treatment of the cytogenetic test were done. ANOVA was performed as before, with the response variables modelled using the interaction between exposure duration and the different plant extract concentrations. In this analysis, we were looking at how duration and increased concentration of SO extracts can treat or mitigate cytotoxicity caused by the SB. Levene’s test was used to check for homogeneity of variance, and Shapiro-Wilk’s test and visual inspection of the models’ residuals were applied to assess normality (Zuur, 2010).

For Correlation analyses, data were first grouped by treatment category (Single or Combined), and each concentration was considered a replicate (n = 3 per treatment). Within each treatment, pairwise associations among biological endpoints (root length, mitotic index, chromosome aberrations, mutation frequency, and polymorphism percentages) were examined using Pearson’s product–moment correlation coefficient (r), which measures the strength and direction of linear relationships (Zar, 2013). Correlation analyses are frequently applied in *Allium cepa* assays to explore mechanistic links between cytogenetic and growth endpoints (Barman and Ray, 2023; Mohammed et al., 2023). Significance was assessed with two-tailed p-values. Because multiple endpoint comparisons were conducted, Benjamini–Hochberg false discovery rate (FDR) correction was applied to control for type I error inflation (Benjamini and Hochberg, 1995). Adjusted p-values ≤0.05 were considered statistically significant. Heatmaps of correlation matrices were produced for visual inspection of patterns. Only results that remained significant after FDR adjustment are reported in the main text. This approach is consistent with recent genotoxicity studies employing the *A. cepa* test, where endpoint-to-endpoint correlations are used to demonstrate coherence among growth inhibition, mitotic suppression, and cytogenetic damage (Barman and Ray, 2023; Mohammed et al., 2023). Although the small number of concentrations limits power, treating concentration levels as replicates has been accepted in exploratory correlation analyses in similar assays (Barman and Ray, 2023).

The mutation frequency was determined according to Qari (Qari, 2016), using the formula;

$$\text{Mutation frequency }\left(\text{MF}\right)=\left(\frac{\text{number}\;\text{of}\;\text{cells}\;\text{with}\;\text{abnormal}\;\text{division}}{\text{total}\;\text{number}\;\text{of}\;\text{cells}}\times 100\right)/\text{mitotic index}$$

## Results

### Determination of EC_50_ of *Sonchus oleraceus*

**FIGURE 2:**
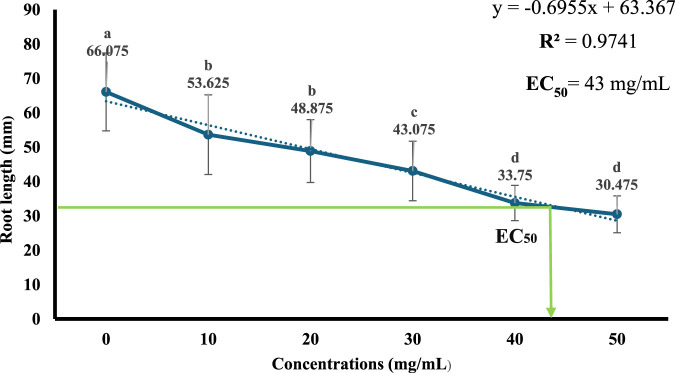
Linear regression for root length percentages of *A. cepa* after treatment with different concentrations of *Sonchus oleraceus* leaves extracts, and determination of EC50. Error bars show the standard deviation of the mean (±SD), (n = 40). Significant differences between groups are indicated by different letters (a–d) above the data points (P ≤ 0.05, ANOVA followed by Tukey’s test).

Figure 2 indicated that the aqueous extracts of *S. oleraceus* leaves inhibited *A. cepa* root length at all concentrations (10, 20, 30, 40, and 50 mg/mL). Then, the median effective concentration (EC_50_) values of the aqueous extract were determined to be 43 mg/mL (50%).

### Roots growth inhibition by *Sonchus oleraceus* leaves extract

**FIGURE 3:**
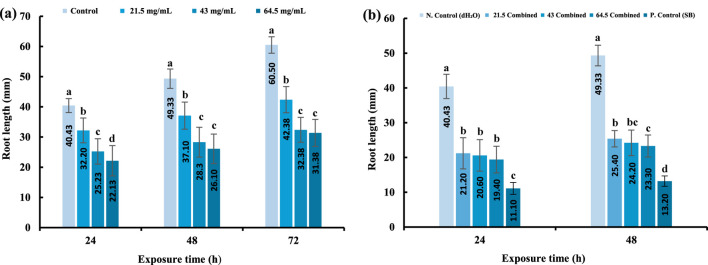
Root length after treatment with aqueous extracts of *Sonchus oleraceus.* **(a)** Single treatments at different concentrations across the exposure times (24, 48, and 72 h). **(b)** Combined treatments (SB + *Sonchus oleraceus*) at different concentrations across the exposure times (24 and 48 h). Error bars represent the mean ± SD (n = 40). Significant differences between groups are indicated by different letters (a–d) above the bars (P ≤ 0.05, ANOVA followed by Tukey’s test).

Figure 3a illustrates the relationships between the root length of *A. cepa* and the impact of different concentrations of aqueous extract of *S. oleraceus* and exposure periods, compared to the control. Figure 3a revealed that the average length of roots decreased progressively and significantly (P ≤ 0.05) as the concentrations and exposure periods increased for all the treatments (21.5, 43, and 64.5 mg/mL). The average root length was 25.23, 28.1, and 32.38 mm, respectively, for 24, 48, and 72 h of exposure to the concentration EC_50_ (43 mg/mL). Conversely, the roots of control had average lengths of 40.43, 49.33, and 60.5 mm, respectively, for 24, 48, and 72 h. Further, it was observed that the average root length decreased with increasing concentration and reached the highest decrease after exposure to the highest concentration of *S. oleraceus* EC_75_ extract (64.5 mg/mL), which amounted to 22.13, 26.1, and 31.38 mm for 24, 48, and 72 h compared to the control group. This suggests a reverse relationship between the concentrations of *S. oleraceus* aqueous extract and the inhibition of cell division in the roots of *A. cepa.*

### Determination of EC_50_ of sodium benzoate

**FIGURE 4:**
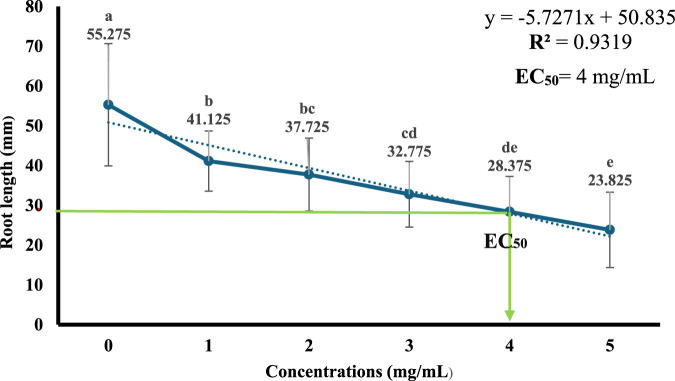
Linear regression for the percentage of root length of *A. cepa* after treatment with different concentrations of sodium benzoate, and determination of EC_50_. Error bars show the standard deviation of the mean (±SD), (n = 40). Significant differences between groups are indicated by different letters (a–e) above the data points (P ≤ 0.05, ANOVA followed by Tukey’s test).

Figure 4 demonstrated that Sodium benzoate decreased the root length *of A. cepa* at all concentrations (1, 2, 3, 4, and 5 mg/mL). Then, the median effective concentration (EC_50_) values for Sodium benzoate were established at 4 mg/mL (50%).

### Roots growth inhibition by combined treatments of *Sonchus oleraceus* leaves extract (SO) and sodium benzoate (SB) in *A. cepa* roots

Figure 3b illustrates the role of the combined treatment with *S. oleraceus* in mitigating the toxicity caused by sodium benzoate on *A. cepa* roots (p ≤ 0.05) by increasing root length. The average root lengths treated with different concentrations of *S. oleraceus* as a combined treatment were compared to the root average of root lengths treated with SB in positive control. The average root length decreased to 11.1 and 13.2 mm after 24 and 48 h in positive control. In contrast, root lengths increased in the combined treatment, especially at the lowest concentration of 21.5 mg/mL, reaching 21.1 and 25.4 mm, respectively. However, high concentrations of *S. oleraceus* and extended exposure durations led to greater root inhibition than the negative control (dH_2_O) and lower than the positive control (SB), indicating that the plant extract partially alleviates SB toxicity; at the same time, it does not completely eradicate it.

### Cytogenetic effects of *Sonchus oleraceus* leaves as a single treatment on *A. cepa* meristematic cells

**FIGURE 5:**
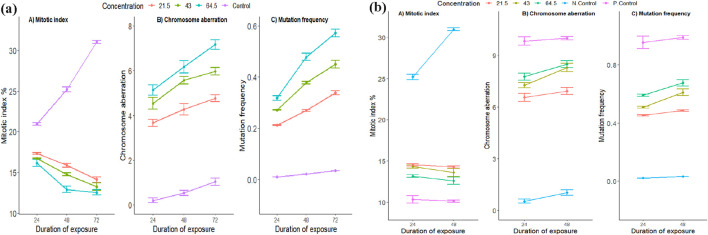
Effects of different concentrations of plant extract *Sonchus oleraceus* on cell division and genetic stability at different times. **(a)** Single treatment (21.5, 43, and 64.5 mg/mL) and control (dH_2_O): Mitotic index, mutation frequency, and Chromosome aberration. **(b)** combined treatment (21.5, 43, and 64.5 mg/mL) compared to negative control (dH_2_O) (N. control) and positive control (SB) (P. control): Mitotic index, mutation frequency, and Chromosome aberration. Error bars represent the mean’s standard deviation (±SD). For each treatment and exposure duration, 3,000 cells from 9 separate roots were analyzed (n = 3,000/9). (P ≤ 0.05, ANOVA followed by Tukey’s test).

Following the preparation of the microscope slide, 3,000 cells were enumerated per treatment (mean of about 1,000) at three intervals: 24, 48, and 72 h. *S. oleraceus* aqueous extracts and sodium benzoate were used as single and combined treatments. In combined treatments with the roots of distilled water as the negative control (dH_2_O) and roots treated with 4 mg/mL of SB transferred to distilled water used as a positive control. The different concentrations of *S. oleraceus* plant demonstrated varying effects on the mitotic index depending on the duration of exposure, as indicated by the significant interaction between duration and concentration (P ≤ 0.05), this suggests that, compared to the control group, the plant extract at different concentrations induced cytotoxic effects, evidenced by a decreased mitotic index in the root tips compared to the control (Figure 5a). The pairwise comparisons revealed that with increasing duration of exposure, the root tip exhibited signs of cytotoxicity, as indicated by the reduced mitotic index (Table 2; Figure 5a). Notably, after 24 h, the mitotic indices across all concentrations were significantly lower than that of the control. As the exposure time increased to 48 h and 72 h, the trend continued, with significant decreases in the mitotic index at all concentrations compared to the control group (Table 2). This indicates an accumulated effect on the mitotic index, resulting in progressively lower cell division rates with increasing concentration and duration (Figure 5a).

**TABLE 2:** Mitotic index of divided meristem cells of *A. cepa* root after exposure to aqueous extract of *Sonchus oleraceus* leaves for different exposure times.

ET (h)	Con. (mg/mL)	TCC	TCD	MI	CA%	MF
24	Control	3000	628	20.93 ± 0.16^a^	0.20 ± 0.10^a^	0.01 ± 0.00^a^
	21.5	3000	520	17.33 ± 0.09^b^	3.67 ± 0.32^b^	0.21 ± 0.00^b^
	43	3000	501	16.70 ± 0.05^bc^	4.53 ± 0.32^c^	0.27 ± 0.00^c^
	64.5	3000	485	16.16 ± 0.45^c^	5.13 ± 0.29^d^	0.32 ± 0.01^d^
48	Control	3000	756	25.20 ± 0.31^a^	0.53 ± 0.15^a^	0.02 ± 0.00^a^
	21.5	3000	476	15.87 ± 0.23^b^	4.27 ± 0.40^b^	0.27 ± 0.00^b^
	43	3000	443	14.77 ± 0.20^c^	5.57 ± 0.23^c^	0.38 ± 0.01^c^
	64.5	3000	387	12.90 ± 0.38^d^	6.17 ± 0.53^d^	0.48 ± 0.01^d^
72	Control	3000	930	31.00 ± 0.16^a^	1.03 ± 0.06^a^	0.03 ± 00^a^
	21.5	3000	423	14.10 ± 0.33^b^	4.77 ± 0.23^b^	0.34 ± 0.01^b^
	43	3000	398	13.26 ± 0.46^c^	5.97 ± 0.13^c^	0.45 ± 0.02^c^
	64.5	3000	376	12.53 ± 0.33^d^	7.17 ± 0.25^d^	0.57 ± 0.02^d^

ET(h): Exposure time (hour), Con. (mg/mL): Concentration mg/mL (21.5 = EC_25_; 43 mg/mL = EC_50_; 64.5 mg/mL = EC_75_ for *Sonchus oleraceus*, and 4 mg/mL = EC_50_ for sodium benzoate), TCC: total cells counted, TCD: total cells division, MI %: Mitotic index % ± SD, CA%: Chromosome aberration ±SD, MF: Mutation frequency ±SD, (SD): standard deviation; (a-d): Significant differences between groups are indicated by different letters next to the numbers. For each treatment and exposure duration, 3,000 cells from 9 separate roots were analyzed (n = 3,000/9).

**FIGURE 6:**
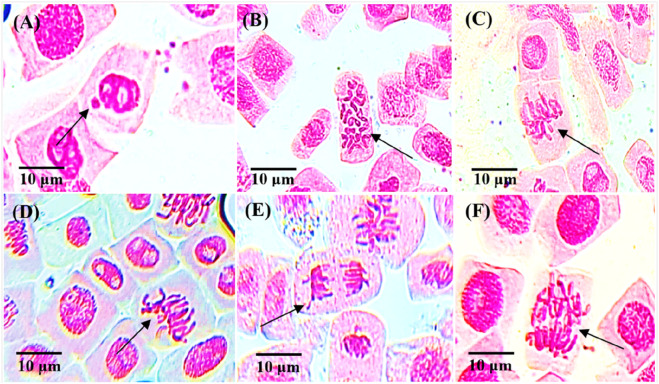
Chromosomal aberrations in *A. cepa* meristematic cells after treatment for 24, 48, and 72 h with different concentrations of aqueous extract of *Sonchus oleraceus* leaves as a single treatment, using a light microscope at ×40 magnification, scale bar = 10 μm; arrows indicate abnormalities: **(A)** micronuclei cell in interphase (64.5 mg/mL for 72 h), **(B)** c-metaphase (21.5 mg/mL for 24 h), **(C)** disturbance (43 mg/mL for 72 h), **(D)** sticky chromosome in metaphase (43 mg/mL for 72 h), **(E)** vagrant chromosome in telophase (64.5 mg/mL for 48 h), **(F)** sticky chromosome in anaphase (64.5 mg/mL for 48 h). For each treatment and exposure duration, 3,000 cells from 9 separate roots were analyzed (n = 3,000/9).

The genotoxicity effect of *S. oleraceus* plant extract and duration of exposure, there was a significant effect of the interactions between duration and concentrations on mutation frequency and chromosome aberration (P ≤ 0.05). Both the different concentrations of plant extract and the duration of the exposure to each concentration induced higher chromosome aberration and mutation frequency compared to the control group (Figure 5a; Table 2). Table 2 and Figure 6 present the results of chromosomal abnormalities. The percentage and types of mitotic chromosomal aberrations induced by treatment with an aqueous leaf extract of *S. oleraceus*, as a single treatment, included micronuclei, c-metaphase, disturbance, vagrant, and stickiness.

### Cytogenetic effects of *Sonchus oleraceus* leaves with sodium benzoate as combined treatments on *A. cepa* meristematic cells (SO + SB)

To assess if *S. oleraceus* could mitigate the adverse effect of SB as a combined treatment, root tips were first treated with SB for 24 h and then treated with *S. oleraceus* at different concentrations for 24 and 48 h. The mitotic index, chromosomal aberration, and mutation frequency of roots subjected to different concentrations of *S. oleraceus* combined treatment (p ≤ 0.05) were compared to those of the positive control (SB). *S. oleraceus* treatment alleviated the adverse impacts on the mitotic index, while both chromosomal aberration and mutation frequency were significantly lower (p ≤ 0.05) than in the positive control (SB) (Table 3; Figure 5b). For the positive control (SB), the chromosomal aberration and mutation frequency at 48 h were 10.00 and 1.03, respectively, whereas for the combined treatment with *S. oleraceus*, they were recorded as 6.93 and 0.45 at 21.5 mg/mL, 8.30 and 0.61 at 43 mg/mL, and 8.50 and 0.68 at 64.5 mg/mL (Table 3; Figure 5b). Although higher concentrations of *S. oleraceus* did not further reduce aberrations or mutations, they slightly increased them with longer exposure durations, leading to worse outcomes compared to the negative control (dH_2_O) group, which recorded values of 0.53 and 1.03. Nevertheless, higher concentrations of the combined treatment with *S. oleraceus* and prolonged treatment durations were substantially less toxic to root cells than the positive control (SB), indicating that the plant extract partially alleviates SB toxicity.

**TABLE 3:** Mitotic index of divided meristem cells of *A. cepa* root after exposure to combined treatment compared negative control and positive control.

ET (h)	Con. (mg/mL)	TCC	TCD	MI	CA%	M inh.%	MF
24	N. Control (dH2O)	3000	756	25.20 ± 0.31^a^	0.53 ± 0.15^a^	-	0.02 ± 0.00^a^
	21.5	3000	436	14.53 ± 0.12^b^	6.57 ± 0.41^b^	35.12	0.45 ± 0.00^b^
	43	3000	430	14.33 ± 0.18^b^	7.27 ± 0.50^c^	24.37	0.53 ± 0.01^c^
	64.5	3000	395	13.17 ± 0.16^c^	7.77 ± 0.25^d^	22.22	0.59 ± 0.01^d^
	P. Control (Sodium benzoate)	3000	**310**	**10.33** ± **0.44** ^d^	**9.83** ± **0.19** ^e^	-	**0.96** ± **0.04** ^e^
48	N. Control (dH2O)	3000	930	31.00 ± 0.16^a^	1.03 ± 0.06^a^	-	0.03 ± 0.00^a^
	21.5	3000	428	14.27 ± 0.15^b^	6.93 ± 0.14^b^	34.20	0.49 ± 0.01^b^
	43	3000	408	13.60 ± 0.49^c^	8.30 ± 0.37^c^	18.96	0.61 ± 0.02^c^
	64.5	3000	378	12.60 ± 0.43^d^	8.50 ± 0.26^c^	16.73	0.68 ± 0.02^d^
	P. Control (Sodium benzoate)	3000	**304**	**10.13** ± **0.13** ^e^	**10.00** ± **0.33** ^d^	-	**1.03 ± 0.01^e^**

ET(h): Exposure time (hour), Con. (mg/mL): Concentration mg/mL (21.5 = EC25; 43mg/mL = EC50; 64.5 mg/mL = EC75 for Sonchus oleraceus), (N. control): Negative control, (P. control): Positive control (4 mg/mL SB), (TCC): total cells counted, (TCD): total cells division, (MI %): Mitotic index % ± SD, (CA%): Chromosome aberration ±SD, (M inh.%): Mutagenicity inhibition %, MF: Mutation frequency ±SD, (SD): standard deviation; (a-e): Significant differences between groups are indicated by different letters next to the numbers. Bolded values indicate the positive control. For each treatment and exposure duration, 3,000 cells from 9 separate roots were analyzed (n = 3,000/9).

**FIGURE 7:**
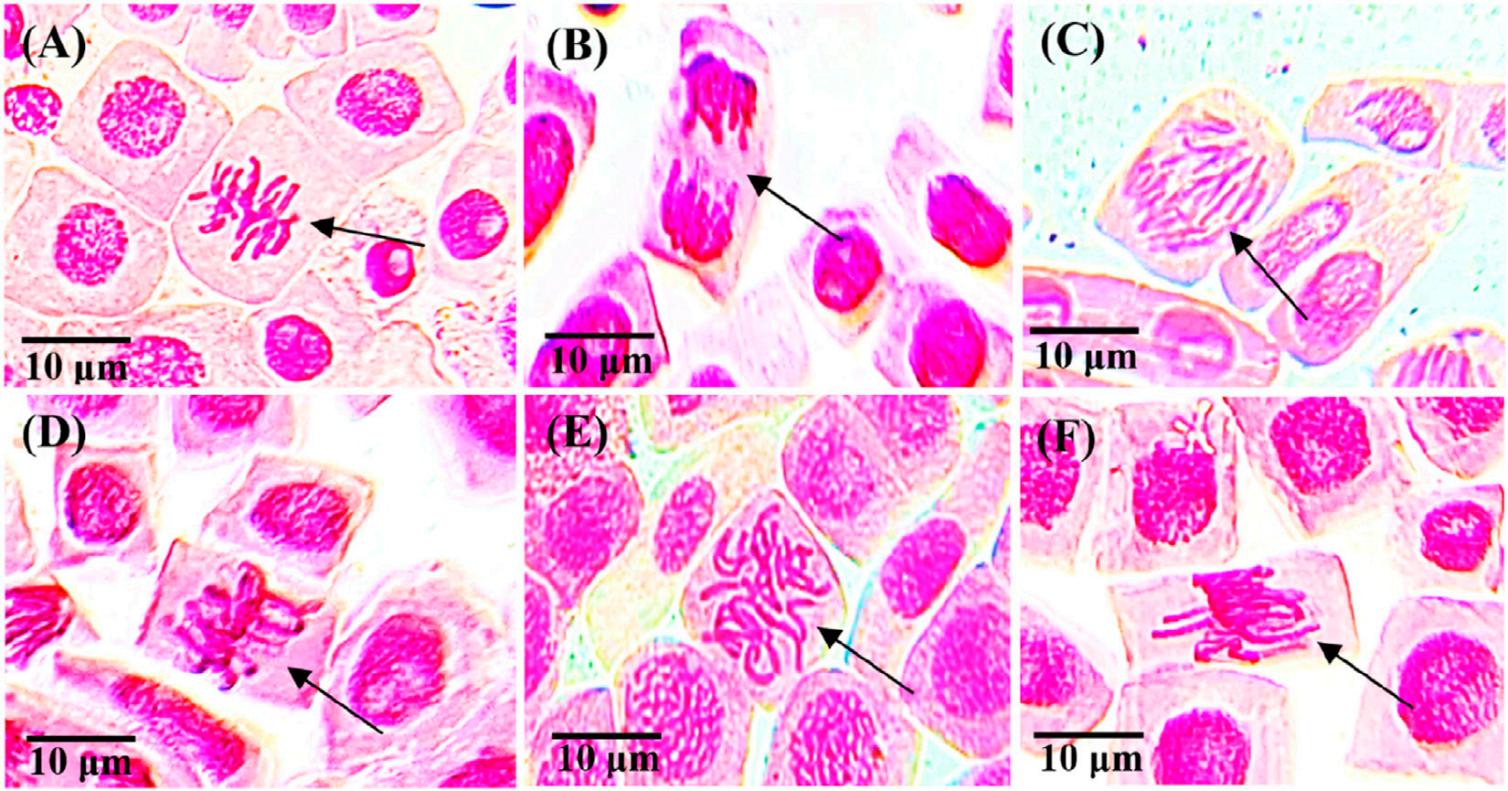
Normal and abnormal chromosomes in *A. cepa* meristematic cells after combined treatment for 24, 48, and 72 h with different concentrations of combined treatment, using a light microscope at ×40 magnification, scale bar = 10 μm; arrows indicate abnormalities: **(A)** normal metaphase (21.5 mg/mL, 24 h), **(B)** normal telophase (21.5 mg/mL, 48 h), **(C)** bridge in anaphase (43 mg/mL, 48 h), **(D)** normal metaphase (43 mg/mL, 24 h), **(E)** c-metaphase (64.5 mg/mL, 24 h), **(F)** sticky chromosome (64.5 mg/mL, 72 h). For each treatment and exposure duration, 3,000 cells from 9 separate roots were analyzed (n = 3,000/9).

Figure 7 shows images of cells at normal and abnormal phases of the mitotic division after combined treatment with different concentrations of *S. oleraceus*. By calculating the percentage of inhibition, it is possible to confirm the inhibitory activity of the chromosomal aberrations caused by SB. The percentage of mutagenicity inhibition of chromosomal aberrations at the concentration of 21.5 mg/mL of combined treatment was 35.12% at 24 h and 34.20% at 48 h (Table 3). This inhibition in chromosomal aberrations was determined to be significant. The extent of antimutagenicity at this specific concentration was moderate, as the percentage of inhibition was greater than 25%. The results showed that the combined treatment of aqueous extract of *S. oleraceus* had a moderate ability to reduce the toxicity of the direct-acting mutagen (SB) at a precise concentration but did not eliminate it entirely.

### Molecular analysis

Molecular tests were carried out with the different concentrations of *S. oleraceus* as a single and combined treatment compared to negative control (dH_2_O) and positive control (Sodium benzoate) for the intermediate exposure period (48 h).

### Molecular analysis of extracted DNA from *A. cepa* roots treated by *Sonchus oleraceus* extract as single and combined treatments with SB

Molecular data of the markers for each of the four primers used (ISSR-UBS-811, ISSR-418, ISSR-HB12, and ISSR-MAO) showed 178 bands after gel electrophoresis analysis (Supplementary Figures S2, S4, S6, S8). The generated bands exhibited a range of lengths, spanning from 193 to 3,076 base pairs (Tables 4–7). The results showed that the total of polymorphism ratio resulting from single (*S. oleraceus* alone) and combined treatment (Sodium benzoate (SB)+ *S. oleraceus*) in the four primers (HB12, 418, UBC-811, and MAO) was 81.81%, 100%, 83.83%, and 84.61%, respectively, compared to the negative control (dH_2_O).

**TABLE 4:** ISSR-HB12 data analysis export from gel electrophoresis sample treated with different concentrations of *Sonchus oleraceus* extract as a single and combined treatment compared to negative control (dH_2_O) for 48 h.

No.	MW (bp)	N. Control (dH_2_O)	Single treatment *S. oleraceus* (mg/mL)			P. Control (SB)	Combined treatment (mg/mL)			Band type
			21.5	43	64.5	4	4 + 21.5	4 + 43	4 + 64.5	
1	1389.93	1	1	1	1	0	1	0	1	P
2	1202.47	0	1	1	0	0	1	0	0	P
3	889.46	1	1	1	1	1	1	1	1	M
4	659.70	1	1	1	1	0	1	0	1	P
5	586.48	0	0	0	0	0	0	1	1	P
6	517.39	1	1	1	1	1	1	1	1	M
7	487.05	0	0	1	0	1	0	0	0	P
8	410.64	1	1	1	1	1	1	1	0	P
9	361.69	0	0	0	1	1	0	0	0	P
10	322.37	1	1	1	0	0	0	1	0	P
11	221.34	1	1	0	0	0	0	1	0	P
Total		7	8	8	6	5	5	6	5	50
Polymorphism %		-	**12.5%**	**33.33%**	**37.5%**	**66.66%**	**37.5%**	**37.5%**	**50%**	-
Total Polymorphism %		**81.81%**								

(NO): number of locus; (MW): molecular weight; (N. Control): negative control; (P. Control): positive control; (SB): Sodium benzoate (P): polymorphic bands, (U): unique bands, (M): monomorphic bands. Bolded values indicate the percentage of the polymorphism for each concentration and the total polymorphism percentage.

**TABLE 5:** ISSR-418 data analysis export from gel electrophoresis sample treated with different concentrations of *Sonchus oleraceus* extract as a single and combined treatment compared to negative control (dH_2_O) for 48 h.

NO.	MW (bp)	N. Control (dH_2_O)	Single treatment *S. oleraceus* (mg/mL)			P. Control (SB)	Combined treatment (mg/mL)			Band type
			21.5	43	64.5	4	4 + 21.5	4 + 43	4 + 64.5	
1	3076.61	0	0	0	0	1	0	0	0	U
2	2022.75	0	0	0	0	1	0	0	0	U
3	1641.78	0	0	0	1	0	0	1	1	P
4	1377.18	0	0	0	0	1	0	0	0	U
5	1078.16	1	1	1	1	0	0	0	1	P
6	941.57	1	1	1	0	1	0	0	0	P
7	842.07	0	0	0	0	0	1	1	1	P
8	694.40	0	0	0	0	1	0	0	0	U
9	664.94	1	1	0	0	0	1	1	0	P
10	616.10	0	0	0	0	1	0	0	1	P
11	551.66	0	1	1	1	0	0	0	0	P
12	386.69	1	1	1	1	0	1	1	1	P
13	321.01	1	0	0	1	0	0	0	0	P
Total		5	5	4	5	6	3	4	5	37
Polymorphism %		-	**33.33%**	**50%**	**57.14%**	**90%**	**66.66%**	**71.43%**	**75%**	-
Total Polymorphism %		**100%**								

(NO): number of locus; (MW): molecular weight; (N. Control): negative control; (P. Control): positive control; (SB): Sodium benzoate (P): polymorphic bands, (U): unique bands, (M): monomorphic bands. Bolded values indicate the percentage of the polymorphism for each concentration and the total polymorphism percentage.

**TABLE 6:** ISSR-UBC-811 data analysis export from gel electrophoresis sample treated with different concentrations of *Sonchus oleraceus* extract as a single and combined treatment compared to negative control (dH_2_O) for 48 h.

NO.	MW (bp)	N. Control (dH_2_O)	Single treatment *S. oleraceus* (mg/mL)			P. Control (SB)	Combined treatment (mg/mL)			Band type
			21.5	43	64.5	4	4 + 21.5	4 + 43	4 + 64.5	
1	1272.30	0	0	0	0	1	0	0	0	U
2	1078.16	0	0	0	0	1	0	0	0	U
3	955.85	1	1	0	1	0	0	1	0	P
4	884.98	0	0	0	0	1	0	0	0	P
5	742.80	1	1	1	1	1	1	1	1	M
6	642.55	0	1	1	1	1	1	1	1	P
7	528.85	1	1	1	1	1	1	1	1	M
8	447.21	0	0	0	0	1	0	0	0	P
9	412.96	1	1	1	0	0	1	0	0	P
10	343.49	0	0	0	0	0	0	1	0	P
11	300.00	1	1	1	1	0	1	1	1	P
12	213.22	1	0	0	1	0	0	0	0	P
Total		6	5	5	6	7	6	6	4	45
Polymorphism%		-	**28.57%**	**42.86%**	**28.57%**	**81.81%**	**42.86%**	**50%**	**57.14%**	-
Total Polymorphism%		**83.83%**								

(NO): number of locus; (MW): molecular weight; (N. Control): negative control; (P. Control): positive control; (SB): Sodium benzoate (P): polymorphic bands, (U): unique bands, (M): monomorphic bands. Bolded values indicate the percentage of the polymorphism for each concentration and the total polymorphism percentage.

**TABLE 7:** ISSR-MAO data analysis export from gel electrophoresis sample treated with different concentrations of *Sonchus oleraceus* extract as a single and combined treatment compared to negative control (dH_2_O) for 48 h.

NO.	MW (bp)	N. Control (dH_2_O)	Single treatment *S. oleraceus* (mg/mL)			P. Control (SB)	Combined treatment (mg/mL)			Band type
			21.5	43	64.5	4	4 + 21.5	4 + 43	4 + 64.5	
1	1445.94	0	0	0	0	1	0	0	0	U
2	1171.21	1	0	0	1	0	0	0	0	P
3	924.02	1	1	0	0	0	0	0	0	P
4	815.86	0	1	1	1	1	0	0	0	P
5	684.75	1	1	1	1	1	1	1	1	M
6	640.98	0	0	0	0	1	0	0	0	U
7	554.90	1	0	1	1	0	1	0	0	P
8	457.31	1	1	1	1	1	1	1	1	M
9	363.42	0	0	0	0	1	1	1	0	P
10	346.41	1	1	0	0	0	0	0	1	P
11	314.73	0	0	1	0	1	0	0	1	P
12	253.37	1	1	1	1	0	1	1	0	P
13	193.36	0	0	0	0	1	0	0	0	U
Total		7	6	6	6	8	4	4	4	46
Polymorphism%		-	**37.5%**	**55.55%**	**37.5%**	**84.62%**	**50%**	**62.5%**	**62.5%**	-
Total Polymorphism%		**84.61%**								

(NO): number of locus; (MW): molecular weight; (N. Control): negative control; (P. Control): positive control; (SB): Sodium benzoate (P): polymorphic bands, (U): unique bands, (M): monomorphic bands. Bolded values indicate the percentage of the polymorphism for each concentration and the total polymorphism percentage.

**FIGURE 8:**
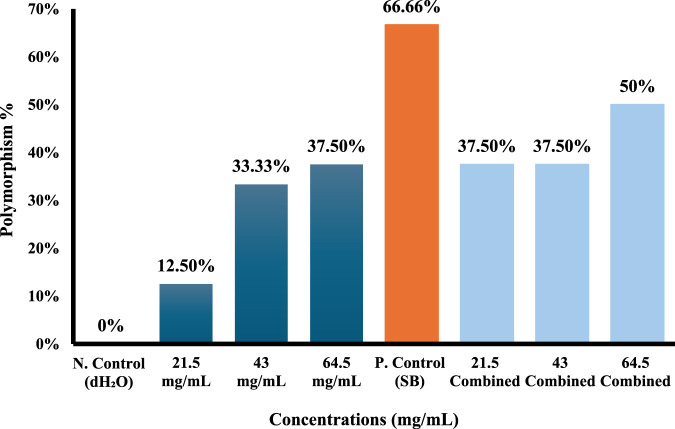
ISSR-HB12 polymorphism % from different concentrations of *Sonchus oleraceus* extract as a single treatment (*Sonchus oleraceus* alone) and combined treatment (Sodium benzoate (SB) (4 mg/mL) + *Sonchus oleraceus*) compared to (dH_2_O) as negative control (N. control) and (SB) as positive control (P. control) for 48 h.

The ISSR-HB12 primer observed polymorphic bands of DNA samples with various concentrations of *S. oleraceus* (21.5, 43, and 64.5 mg/mL) as a single and combined treatment and the negative control (dH_2_O) and positive control (SB) bands for 48 h. The number of ISSR bands that disappeared was greater at higher *S. oleraceus* concentrations (Table 4; Figure 8), with bands at locus 10 and 11 of molecular size from about 322 to 221 bp were shown to disappear after being exposed to 64.5 mg/mL of *S. oleraceus* extract alone, the polymorphism ratio for this concentration was 37.5%.

The alterations in density bands and polymorphism bands generated by the SB effect in *Allium cepa* cells were diminished and partially rectified relative to the negative control. For example, four bands at locus 1, 4, 10, and 11 with molecular sizes (1389, 659, 322, and 221 bp) disappeared, and the polymorphism ratio was 66.66% in the positive control (SB).

Conversely, the number of disappearing bands diminished at 21.5 mg/mL in combined treatment, decreased to two bands at locus 10 and 11 with molecular sizes ranging between 322 and 221 bp, and the polymorphism ratio decreased to 37.5%. Nonetheless, the quantity of disappearing bands was noted to rise with higher concentrations (Table 4; Figure 8).

**FIGURE 9:**
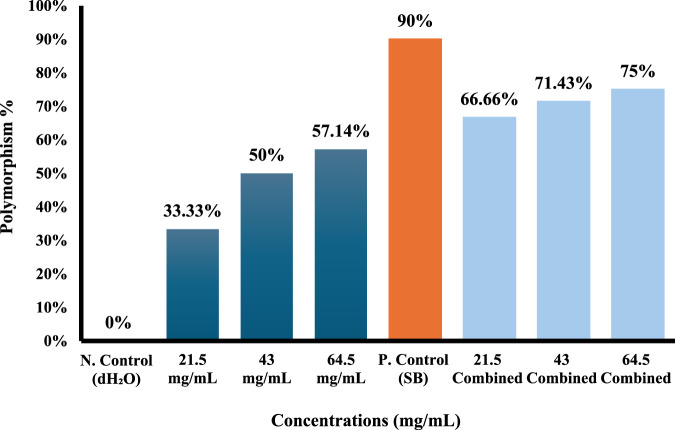
ISSR-418 polymorphism % from different concentrations of *Sonchus oleraceus* extract as a single treatment and combined treatment (SO+SB) compared to (dH_2_O) as negative control (N. control) and (SB) as positive control (P. control) for 48 h.

ISSR-418 fingerprints exhibited significant disparities between control and exposure treatments, marked by distinct alterations in the number and intensity of amplified DNA bands, yielding a total polymorphism ratio of 100% (Table 5; Figure 9).

The decrease in band intensity was significantly evident for *Allium cepa* exposed to 43 and 64.5 mg/mL of *S. oleraceus* alone; bands at locus 9 and 13 of molecular sizes of about 664 and 321 bp disappeared in 43 mg/mL, and the polymorphism ratio reached 50%. The bands disappeared at 64.5 mg/mL concentration at locus 6 and 9 with molecular sizes 941 and 664 bp, and the ratio of polymorphism increased with increasing concentration, reaching 57.14%.

The combined treatments reduced the number of polymorphic bands formed by SB. The polymorphism ratio in the positive control group (SB) was 90%, the number of ISSR bands gained was more pronounced in it, with five bands gained in locus number one (3,076 bp), number two (2,022 bp), number four (1,377 bp), number eight (694 bp), and number ten (616 bp). On the contrary, at the lowest concentration of 21.5 mg/mL in the combined treatment, the number of gained bands decreased to one band in locus number 7 (842 bp), and the polymorphism ratio also decreased to 66.66% (Table 5; Figure 9).

However, an increase in the number of acquired bands was observed with higher concentrations at 64.5 mg/mL, where 3 bands were gained in locus numbers 3, 7, and 10 (1641, 842, and 616 bp), respectively, with a polymorphism ratio of 75%.

**FIGURE 10:**
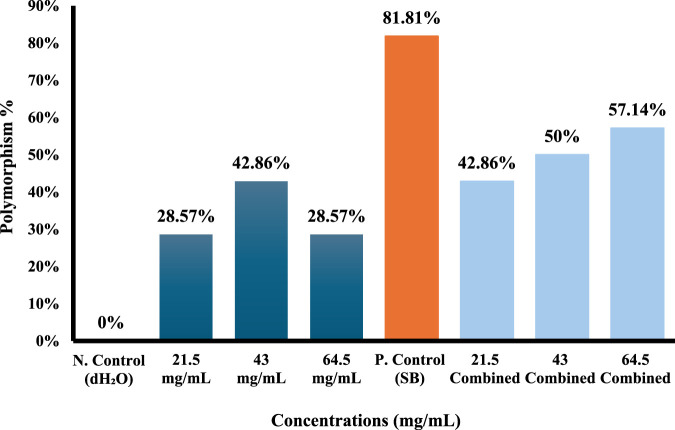
ISSR-UBC-811 polymorphism % from different concentrations of *Sonchus oleraceus* extract as a single treatment and combined treatment (SO+SB) compared to (dH_2_O) as negative control (N. control) and (SB) as positive control (P. control) for 48 h.

The polymorphic bands of another ISSR marker, which is called ISSR-UBS 811, were observed. The number of ISSR bands that disappeared increased as the concentration of *S. oleraceus* increased. After exposure to 43 mg/mL of *S. oleraceus* extract, bands at locus 3 and 12 with molecular sizes of about 955 to 213 bp disappeared, with a polymorphism rate of 42.86% (Table 6; Figure 10).

The changes in band density and polymorphism in *Allium cepa* cells caused by the SB effect were reduced and somewhat fixed by the combined treatments compared to the control. The highest polymorphism ratio was in the positive control (SB) at 81.81% and the number of ISSR bands gained was more pronounced in it; five bands were gained at locus 1 (1,272 bp), 2 (1,078 bp), 4 (884 bp), 6 (642 bp), and 8 (447 bp).

While in the combined treatment, especially at the lowest concentration (21.5 mg/mL), the polymorphism rate decreased to 42.86%, and the number of gained bands diminished to one band at locus number 6 (642 bp). However, an increase in the number of bands gained was observed with higher concentrations of *S. oleraceus* as combined treatment but remained closer to negative control than positive control (SB) (Table 6; Figure 10).

**FIGURE 11:**
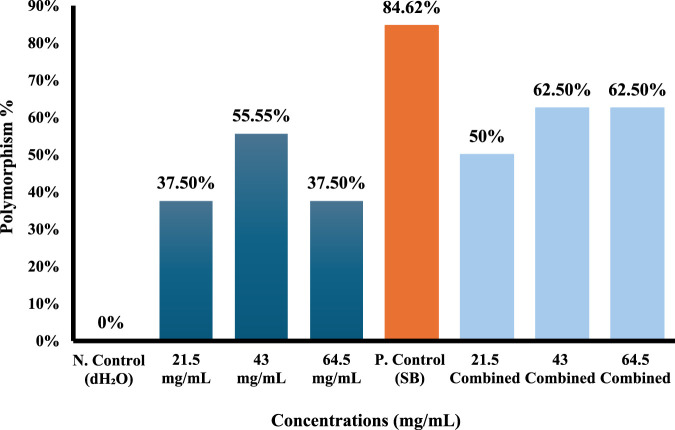
ISSR-MAO polymorphism % from different concentrations of *Sonchus oleraceus* extract as a single treatment and combined treatment compared to (dH_2_O) as negative control (N. control) and (SB) as positive control (P. control) for 48 h.

According to the results of the bands shown by the MAO marker, the polymorphism ratio increased to 55.55% after exposure to 43 mg/mL of *S. oleraceus* extract alone; bands were lost at locus numbers 2, 3, and 10 with molecular sizes 1,171, 924, and 346 bp, respectively; also, two bands were gained at locus numbers 4 and 11 (815 and 314 bp) (Table 7; Figure 11).

In comparison to the positive control (SB), the changes in density bands and polymorphism bands induced by SB in *Allium cepa* cells were reduced and partially reversed in the combined treatment. The polymorphism ratio in the positive control (SB) group was 84.62%, and the positive control (SB) group lost five bands at loci 2 (1,171 bp), 3 (924 bp), 7 (554 bp), 10 (346 bp), and 12 (253 bp).

In contrast, at the lowest concentration (21.5 mg/mL) in the combined treatment, the polymorphism ratio decreased to 50%, and the number of lost bands decreased to only three bands at locus numbers 2 (1,171 bp), 3 (924 bp), and 10 (346 bp). Nonetheless, a rise in the quantity of vanishing bands was noted with elevated concentrations of *S. oleraceus* as a co-treatment, yet it stayed nearer to negative control than positive control (SB) (Table 7; Figure 11).

The phylogenetic tree diagram of four primers (HB12, 418, UBC-811, and MAO) was used as a marker to show the relationship between single and shared bands at different concentrations of *S. oleraceus* extract and positive control (SB) compared to the negative control (dH_2_O) using UPGMA analysis. The findings revealed four clades exhibiting low genetic variance within a distance range of 0.56–0.83, 0.42–0.64, 0.68–0.81, and 0.44–0.68, respectively, where the type and concentration of treatment determine the relationship between the negative control (dH_2_O) and the treatments (Supplementary Figures S2, S4, S6, S8). The clades that received the single treatment of *S. oleraceus* at the lowest concentration of 21.5 mg/mL of the plant extract, followed by 43 and 64.5 mg/mL, were the closest to the negative control, followed by the least concentration of the combined treatment, 21.5 mg/mL. The distance increases with higher concentrations, and the positive control (SB) was the farthest from the negative control.

### Statistical correlation between different parameters

**FIGURE 12:**
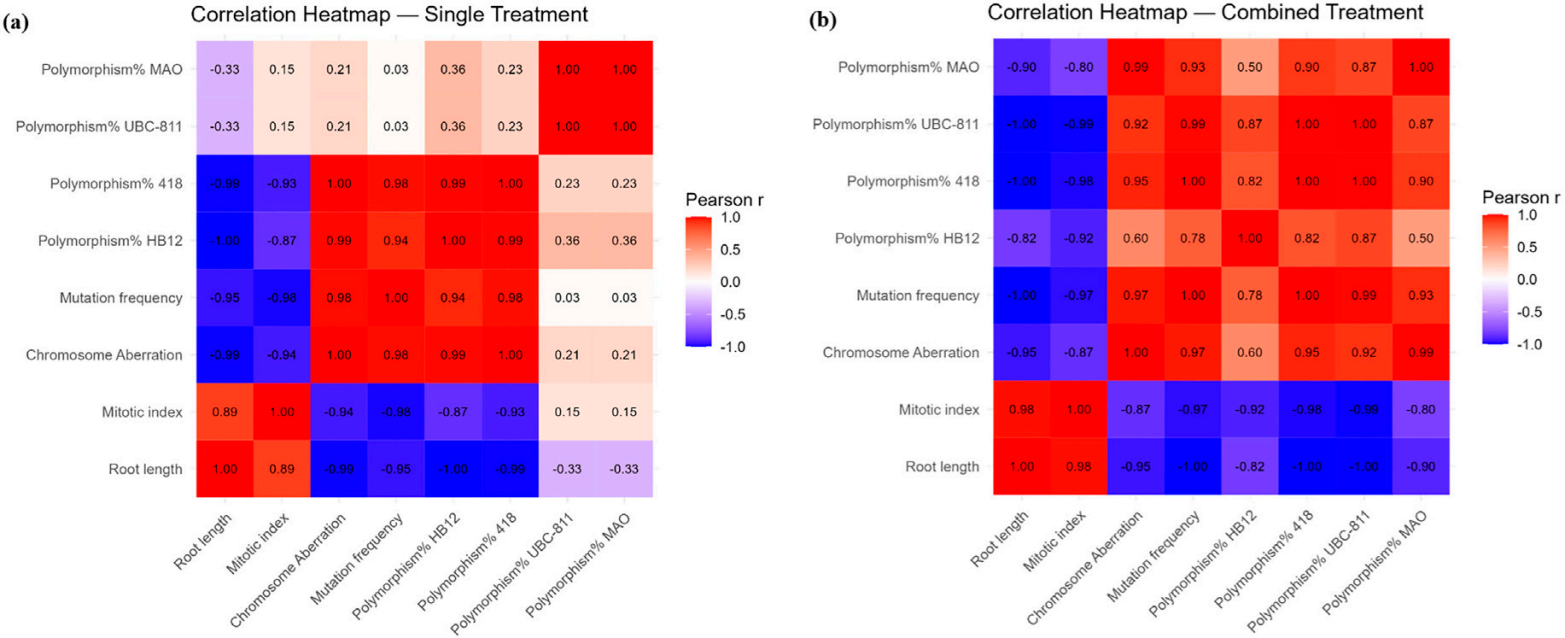
Correlation heatmap illustrating pairwise relationships among cytogenetic (root length, mitotic index, chromosomal abnormalities, mutation frequency) and molecular (ISSR polymorphism%) endpoints in *Allium cepa* under **(a)** Single (SO alone) and **(b)** Combined treatments (SB + SO).

In the *Allium cepa* assay, endpoint–endpoint correlations were examined separately for Single and Combined treatments (n = 3 concentrations per treatment). Across both treatments, a consistent set of strong associations was observed. In the Single treatment, root length was negatively correlated with mutation frequency (r = −0.998, p = 0.044) and with polymorphism% of ISSR-418 (r = −1.000, p = 0.0004). Mutation frequency was positively correlated with polymorphism% 418 (r = 0.998, p = 0.043) (Figure 12). In the Combined treatment, root length again showed negative correlations with mutation frequency (r = −0.998, p = 0.044) and polymorphism% of ISSR-418 (r = −1.000, p = 0.0004), while mutation frequency was positively correlated with polymorphism% 418 (r = 0.998, p = 0.043). Other correlations among endpoints (e.g., mitotic index, chromosome aberrations, polymorphism HB12, UBC-811, MAO) were moderate to high in magnitude but did not reach statistical significance under the limited sample size (Figure 12). These results suggest that inhibition of root growth was closely associated with increased mutation frequency and molecular polymorphism, consistently across treatment conditions.

## Discussion

The potential cellular and molecular effects of the aqueous extract of *Sonchus oleraceus* (SO), as well as its mitigating role against sodium benzoate (SB)-induced cytotoxicity and genotoxicity on *Allium cepa* root cells, were evaluated. The leaf extract of S. oleraceus exhibited a dose-dependent effect on *A. cepa* cells: at lower doses, it moderately decreased sodium benzoate-induced cytotoxicity and genotoxicity, whereas at higher doses, it increased these effects. The findings suggest that the extract’s biological activity is concentration-dependent. The results indicated that the EC_50_ was determined at a concentration of 43 mg/mL, and the higher concentration of SO extract (50 mg/mL) led to a marked decrease in seedling growth (53.88%) compared to the control. This finding suggests that SO extract may interfere with the mitotic division or DNA replication processes, thereby impairing growth and disrupting the triploid endosperm that nourishes the embryo, ultimately leading to malnutrition and cellular death (Dragoeva et al., 2015). The tested concentrations (21.5, 43, and 64.5 mg/mL) cover values below, at, and above the typical human infusion concentration (∼40 mg/mL (Vecchia et al., 2022)), enabling the evaluation of dose-dependent effects in relation to real exposure.

The results demonstrated that single treatment with SO extract significantly decreased root growth of *A. cepa* at all tested concentrations, with the most pronounced effect observed at 64.5 mg/mL (P ≤ 0.05) (Figure 3a). Root growth parameters serve as important indicators for assessing the cytotoxicity and genotoxicity of medicinal plant extracts (Alabi et al., 2022). These findings imply that certain concentrations of the extract exert adverse cytotoxic effects on the meristematic tissues of *A. cepa* roots. This result is consistent with the findings of Saxena et al. (2010) and Khanna and Sharma (2013), who reported that aqueous extracts of various herbal plants inhibited root elongation, suggesting toxic effects on cell division.

Several studies have indicated that natural compounds can mitigate the toxicity induced by SB in animal models (Yassien et al., 2022; Oladele et al., 2020; Kameswari et al., 2023). The concentration of sodium benzoate at 4 mg/mL was experimentally established as the EC50 in the *A. cepa* assay, utilizing root growth measurements after 72 h and linear regression analysis. This concentration signifies a threshold particular to the bioassay rather than a predetermined toxicological limit. For context, regulatory agencies establish the acceptable daily intake (ADI) at 5 mg/kg body weight (Lennerz et al., 2015; Zhang and Ma, 2013); the Brazilian authority ANVISA permits a maximum concentration of 0.05 g/100 mL (500 mg/L) in beverages (Zhang and Ma, 2013), whereas the U.S. FDA designates sodium benzoate as Generally Recognized As Safe (GRAS) and allows its application up to 0.1% by weight in foods and beverages, adhering to Good Manufacturing Practices (Sreenivasan et al., 2023). Therefore, although the EC50 found in this study exceeds typical dietary exposures, it is valuable for defining cytotoxic and genotoxic thresholds in a controlled bioassay, providing important insight into the potential biological effects of sodium benzoate. In the current study, SB alone significantly inhibited root elongation in *A. cepa*; however, co-treatment with SO extract at all concentrations, especially at 21.5 mg/mL, led to a partial recovery of root length. This suggests a concentration-dependent moderate mitigating effect of SO. Acar (2021) similarly demonstrated that SB reduces *A. cepa* germination rate, while co-administration of royal jelly ameliorated this toxicity. Nevertheless, it was observed that higher SO concentrations and longer exposure times increased root growth inhibition, indicating potential toxicity of the extract at elevated doses (Figure 3b). This biphasic behavior supports prior observations that lower concentrations of plant extracts may exert antimutagenic effects, whereas higher concentrations may induce mutagenicity (Pérez-Carreón et al., 2002; Yamanaka et al., 1997; Ene and Osuala, 1990; Hayakawa et al., 1999).

The mitotic index (MI) data further confirmed the cytotoxic effects of SO. Different concentrations of the extract led to a significant reduction in MI across all exposure times (P ≤ 0.05), especially at 64.5 mg/mL after 72 h, which showed the most prominent decrease (Table 2). In line with Chukwujekwu and Van Staden (2014), a reduction in MI reflects the inhibitory impact of phytochemicals on cell proliferation. This inhibition may result from interference with DNA synthesis or disruptions at the G2/M checkpoint, potentially through interactions between extract constituents and DNA, particularly via hydroxyl terminal groups (Oulahal and Degraeve, 2022).

The results also revealed that different concentrations of SO extract caused a significant increase in chromosomal aberrations and mutation frequency compared to the control group (Figure 5a; Table 2). These aberrations suggest that SO extract, especially at higher concentrations, may contain compounds such as alkaloids, flavonoids, or tannins capable of disrupting chromosomal integrity and mitotic progression (Lubini et al., 2008). Importantly, treatment with *S. oleraceus* extract (SO) alleviated the adverse effects of SB on the mitotic index in *A. cepa* cells. The combined treatment groups exhibited significantly higher MI values compared to the positive control (SB), particularly at the lowest concentration (21.5 mg/mL). These findings align with previous studies that reported the concentration-dependent toxicity of SB, which leads to inhibition of cell proliferation and mitotic progression (Zengin et al., 2011; Pongsavee, 2015; Saatci et al., 2016; Lestari et al., 2017; Kumar and Pandey, 2015).

SB treatment also resulted in an increased frequency of chromosomal aberrations and mutations, likely due to oxidative stress caused by glutathione depletion, increased malondialdehyde (MDA) levels, and decreased antioxidant enzyme activities such as SOD and CAT (Acar, 2021; Khoshnoud et al., 2018; Gaur et al., 2018; Yetuk et al., 2014). In contrast, the combined treatments with SO significantly reduced these genotoxic indicators (P ≤ 0.05), particularly at 21.5 mg/mL, which yielded chromosomal aberration percentages of 6.57% and 6.93% at 24 and 48 h, respectively, lower than the values observed in the SB-only group. The chromosomal aberration and mutation frequency values were substantially higher in the positive control compared to all combined treatment groups, demonstrating that SO extract partially mitigates SB-induced toxicity. However, increasing the concentration of SO in the combined treatments did not lead to further reductions in aberrations or mutations; instead, a slight increase was noted with prolonged exposure times, rendering these effects more severe than those in the negative control group (dH_2_O). This outcome supports the notion that phytochemicals may exhibit pro-oxidant behavior at high concentrations, especially in crude extracts where synergistic, antagonistic, or additive interactions among constituents can occur (Pérez-Carreón et al., 2002; Yamanaka et al., 1997; Ene and Osuala, 1990; Hayakawa et al., 1999).

The clastogenic effects observed in *A. cepa* root meristematic cells could be attributed to specific bioactive compounds in SO, such as polyphenols, flavonoids, alkaloids, and tannins, which have been previously shown to cause chromosomal damage under certain conditions. Moreover, when the extract is used in high concentrations or poorly diluted with water, and especially in the presence of reducing agents like vitamin C, there is an increased risk of benzene formation during interaction with SB (Gaur et al., 2018; Medeiros Vinci et al., 2012). These factors may lead to diminished mitigating activity or the conversion of SO constituents into potentially mutagenic metabolites. Conversely, at lower concentrations, the SO extract exhibited antimutagenic properties. This could be due to the ability of the plant’s polyphenols and flavonoids to scavenge reactive oxygen species (ROS), thereby stabilizing cellular structures and preventing mutagenesis. This dual behavior, where an extract shows both genotoxic and antigenotoxic activities depending on the dose, is consistent with the concept of “Janus carcinogens” or dual-role agents (Zeiger, 2003). Therefore, these findings underscore the importance of precise dosage determination for herbal remedies, especially when used in conjunction with food additives like SB.

The antimutagenic potential of SO in this study could be associated with its antioxidant capacity, but these factors were not assessed in the current research. The aqueous leaf extract demonstrated significant inhibition of SB-induced chromosomal aberrations, with inhibition percentages of 35.12% at 24 h and 34.20% at 48 h at 21.5 mg/mL (Table 3). According to Verschaeve and Van Staden (Verschaeve et al., 2008), inhibition values between 25% and 40% are considered moderate, while values above 40% indicate strong antimutagenic activity. Our data thus support the classification of SO as a moderate antimutagen at this concentration. Ferguson (Ferguson, 1994; Ferguson, 2001) proposed that antioxidant compounds may inhibit the genotoxic effects of various mutagens through mechanisms including radical scavenging and DNA repair enhancement. The observed moderate mitigating effects in the combined treatment may also arise from reduced concentrations of SB-reactive components or changes in their chemical profiles when co-administered with SO extract.

The presence of bioactive phytochemicals in SO likely accounts for its dual genotoxic and antigenotoxic effects. Two additional mechanisms may also explain this moderate mitigating behavior: 1. SO extract might adsorb mutagenic compounds in a manner similar to chlorophyllin or hemin, thereby preventing their interaction with DNA (Ferguson et al., 2004; Nogueira et al., 2006), and 2. SO extract may induce DNA glycosylase enzymes, which are involved in excising damaged bases and initiating base excision repair pathways (Steele and Kelloff, 2005).

According to Zietkiewicz et al. (1994), ISSR is a dominant molecular marker technique that amplifies DNA regions located between microsatellite sequences, effectively revealing genomic alterations. This method is frequently employed in plant genetic studies due to its reproducibility and efficiency compared to techniques like RAPD and AFLP (Marri et al., 2002). In the present study, ISSR-PCR analysis confirmed the occurrence of DNA damage in *A. cepa* cells treated with various concentrations of SO extract, especially at the highest concentration (64.5 mg/mL). This damage was evidenced by the disappearance of specific DNA bands, such as those at loci 10 and 11 (322–221 bp) in the ISSR-HB12 primer, which indicated a polymorphism rate of 37.5% (Table 4; Figure 8).

Alterations in band intensity and polymorphism were observed across all four primers used (HB12, 418, UBC-811, and MAO), with the most pronounced changes occurring under higher extract concentrations or in the SB-treated group. Disappearance of bands is typically attributed to deletions or mutations at primer annealing sites, while the appearance of new bands may result from DNA repair mechanisms, replication errors, or chromosomal rearrangements (Bernardes et al., 2015; Enan, 2007). These changes reflect underlying genomic instability and potential genotoxic stress.

Notably, the SB-only group exhibited the highest level of band diversity and polymorphism, particularly with ISSR-418, where a polymorphism rate of 90% was recorded. This indicates substantial genomic disruption and supports earlier cytogenetic observations of SB-induced damage (Table 5; Figure 9). In contrast, co-treatment with SO extract significantly reduced these effects, particularly at the lowest concentration (21.5 mg/mL), where fewer bands disappeared or appeared compared to SB alone. This result suggests that SO has a moderate stabilizing effect on DNA integrity, likely by reducing oxidative damage and enhancing DNA repair pathways (Atienzar et al., 1999; Silva et al., 2008).

According to previous studies, SO comprises polyphenols that may facilitate antioxidant processes. These moderate mitigating effects could be meditated through the activities of polyphenols in the extract may stimulate the Nrf2/ARE pathway, enhancing the expression of antioxidant and cytoprotective genes, including phase II detoxifying enzymes (HO-1, NQO1, GCLC), which could aid in replenishing glutathione levels and sustaining redox homeostasis, thus mitigating ROS accumulation and decreasing DNA strand breaks and chromosomal instability (Dey et al., 2024; Kaurinovic and Vastag, 2019). Previous studies by Abdelhameed et al. (2025) support this, indicating that *S. oleraceus* extract significantly activates the Nrf2/KEAP1/HO-1 pathway in a paracetamol-induced hepatotoxicity model. The activation of this pathway resulted in increased production of antioxidant enzymes and decreased oxidative damage and apoptosis. Additionally, the observed decrease in chromosomal abnormalities at low extract concentrations may lead to the stabilization and enhancement of endogenous antioxidant enzymes (SOD, CAT, and GPx) by phenolic compounds, which are frequently depleted by SB exposure (Vecchia et al., 2022; Kaurinovic and Vastag, 2019). Through redox-sensitive signaling, polyphenols may also affect DNA integrity by modifying repair pathways, including base-excision repair enzymes (DNA glycosylases). This could help to explain why treated roots have fewer DNA damage markers (Sánchez-Aguirre et al., 2024; Jimoh et al., 2011; Alrekabi and Hamad, 2018; Xia et al., 2011; Sal et al., 2023; Pereira-Wilson et al., 2011). The observed biphasic, concentration-dependent response to the established antioxidant/pro-oxidant switch—where low doses mitigated SB-induced genotoxicity while higher doses intensified it—may indicate hormetic effects and a potential dual genotoxic/antigenotoxic behavior (Kaurinovic and Vastag, 2019; Salas-Coronado et al., 2019). At lower concentrations, polyphenols and flavonoids may provide mitigating, antigenotoxic effects; conversely, at higher concentrations, they could function as pro-oxidants, producing reactive oxygen species (ROS) and activating stress-related pathways, including p53 and MAPK (ERK, JNK, p38), which may lead to increased DNA damage and mitotic disturbances (Pereira-Wilson et al., 2011; Salas-Coronado et al., 2019). A tenable dual genotoxic/antigenotoxic mechanism in deciding the mitigating effect outcome of SO is supported by the findings, which point to a concentration-dependent balance between beneficial and perhaps harmful effects (Abdelhameed et al., 2025; Pereira-Wilson et al., 2011; Salas-Coronado et al., 2019).

The phylogenetic analysis of band patterns further demonstrated that SO treatments, particularly at low concentrations, clustered closer to the negative control group than the SB-positive control group, reinforcing the conclusion that SO partially reverses SB-induced genetic damage.

On the other hand, a consistent pattern across both treatments was found by the *A. cepa* correlation analysis, which demonstrated a substantial association between chromosomal damage and root development inhibition. Mutation frequency and polymorphism percentage of ISSR-418 showed a negative correlation with root length, indicating that cytogenetic instability could be the cause of decreased root elongation (Leme and Marin-Morales, 2009; Geirid Allium Test for Screening Chemicals, 1997). Similar to our findings, studies on *A. cepa* subjected to Bisphenol A revealed a significant negative connection between root length and indicators of chromosomal aberrations/mutations, reinforcing the notion that growth inhibition signifies underlying genetic harm (Vujčić Bok et al., 2023; Rybczyńska-Tkaczyk et al., 2023). The robust positive association between mutation frequency and polymorphism percentage of ISSR-418 underscores the sensitivity of ISSR molecular markers in identifying DNA modifications (Figure 12). Genetic diversity studies utilizing ISSR markers in onion populations have shown that ISSR effectively identifies molecular polymorphisms, complementing cytogenetic results (Sudha et al., 2018). The consistency of these correlations under both single and combination treatments implies that the molecular link between growth inhibition and genomic variability is robust, even under different stress situations. Overall, these findings endorse the amalgamation of cytogenetic and molecular biomarkers for a more thorough evaluation of genotoxic potential (Leme and Marin-Morales, 2009; Nicuta et al., 2025).

Although these findings demonstrate the potential of SO extract as a moderate natural antimutagenic agent, it is important to acknowledge certain limitations in the current study. The data were generated exclusively using the *A. cepa* model, which, despite being well-established for genotoxicity testing, may not fully replicate responses observed in mammalian systems. Moreover, the crude extract of SO contains a complex mixture of phytochemicals, making it difficult to determine which specific compounds are responsible for the observed effects. The molecular technique employed (ISSR-PCR), while effective in detecting polymorphisms, does not provide gene-specific expression data. Additionally, it should be highlighted that ISSR analysis in this work was qualitative in nature, and quantitative validation procedures, such as quantitative PCR (qPCR), were not performed.

Future research should focus on *in vivo* studies using mammalian models to validate these results and evaluate the safety profile of SO in higher organisms. Additionally, isolation and characterization of the active constituents within the SO extract and antioxidant tests (e.g., DPPH, FRAP) are necessary to understand their precise molecular mechanisms. Studies exploring the co-effects of SO and SB on human normal and cancer cell lines, coupled with transcriptomic or proteomic analysis, would offer deeper insights into its mitigating potential and toxicological safety.

## Conclusion

This study demonstrates that aqueous extracts of *S. oleraceus* exert a concentration-dependent genotoxic effect on *A. cepa* cells, particularly at high concentrations. SB treatment alone significantly disrupted mitotic activity, increased chromosomal aberrations, and induced genomic instability. Co-treatment with SO extract, especially at 21.5 mg/mL, partially ameliorated these effects, indicating moderate antimutagenic activity. While the current study did not assess antioxidant activity, prior studies suggest that the observed effects could be attributed to the antioxidant potential of SO, which is primarily mediated by its polyphenolic content. Nonetheless, at high concentrations, SO exhibited genotoxicity, underscoring the need for precise dosage determination in traditional and clinical applications.

Although the *A. cepa* model was used in our study, these findings provide the basis for further investigation into similar mitigating effects in more complex biological systems, which could support the development of strategies and methods using dietary antioxidants to mitigate sodium benzoate-induced toxicity. Overall, our findings highlight the complexity of natural plant extracts, which may exert both ameliorative and harmful biological effects depending on dose and context. This study emphasizes the necessity of comprehensive toxicological evaluations for herbal compounds, even those traditionally used. The cytotoxic effects of SB in *A. cepa* underscore the potential concerns warranting further investigation.

## Data Availability

The original contributions presented in the study are included in the article/Supplementary Material, further inquiries can be directed to the corresponding author.

## Glossary

***A. cepa***: *Allium cepa*
**AFLP**: Amplified Fragment Length Polymorphism
**ANOVA**: Analysis of Variance
**bp**: Base pair
**CA**: Chromosomal Aberration
**CAT**: Catalase
**CTAB**: Cetyltrimethylammonium Bromide
**DNA**: Deoxyribonucleic acid
**dNTP**: Deoxynucleotide triphosphates
**dH**_**2**_ **O**: Distlied water
EC_50_: Half maximal effective concentration
**h**: hours
**ISSR**: Inter Simple Sequence Repeat
**MDA**: Malondialdehyde
**MgCl2**: Magnesium Chloride
**MF**: Mutation frequency
**MI**: Mitotic Index
**MN**: Micronucleus
**MW**: Molecular Weight
**Nrf2**: Nuclear factor erythroid 2–related factor 2
**PCR**: Polymerase Chain Reaction
**RAPD**: Random Amplified Polymorphic DNA
**ROS**: Reactive Oxygen Species
**SB**: Sodium Benzoate
**SD**: Standard Deviation
**SO**: *Sonchus oleraceus*
**SCEs**: Sister Chromatid Exchanges
**SOD**: Superoxide Dismutase
**TBE**: Tris Borate EDTA
**TCC**: Total cells counted
**TCD**: Total cells division
**WHO**: World Health Organization

## References

[B1] Abdelhameed M. F. El-Baset M. A. Khattab A. R. Taher R. F. El-Saied M. A. Abd Elkarim A. S. (2025). Hepatoprotective action of Sonchus oleraceus against paracetamol-induced toxicity *via* Nrf2/KEAP-1/HO-1 pathway in relation to its metabolite fingerprint and *in silico* studies. PLoS One 20, e0325782. 10.1371/journal.pone.0325782 40569949 PMC12200742

[B2] Aboul-Maaty N.A.-F. Oraby H.A.-S. (2019). Extraction of high-quality genomic DNA from different plant orders applying a modified CTAB-based method. Bull. Natl. Res. Cent. 43, 25–10. 10.1186/s42269-019-0066-1

[B3] Acar A. (2021). Therapeutic effects of royal jelly against sodium benzoate-induced toxicity: cytotoxic, genotoxic, and biochemical assessment. Environ. Sci. Pollut. Res. Int. 28, 34410–34425. 10.1007/s11356-021-13172-6 33646542

[B4] Afshar M. Moallem S. A. Taheri M. H. Shahsavan M. Sukhtanloo F. Salehi F. (2012). Effect of long term consumption of sodium benzote before and during pregnancy on growth indexes of fetal Balb/c mice. Mod. Care J. 9, 173–180.

[B5] Akwu N. A. Naidoo Y. Singh M. (2019). Cytogenotoxic and biological evaluation of the aqueous extracts of grewia lasiocarpa: an Allium cepa assay. South Afr. J. Bot. 125, 371–380. 10.1016/j.sajb.2019.08.009

[B6] Al-Naqeb G. Zorzi G. Oldani A. Azzalin A. Avesani L. Guzzo F. (2024). Phytochemical profile and *in vitro* cytotoxic, genotoxic, and antigenotoxic evaluation of Cistus monspeliensis L. leaf extract. Int. J. Mol. Sci. 25, 13707. 10.3390/ijms252413707 39769467 PMC11676674

[B7] Alabi O. A. Atanda H. C. Olumurewa J. A. (2022). Cytogenotoxicity of the aqueous extract of parquetina nigrescens leaf using Allium cepa assay. Protoplasma 259, 1417–1425. 10.1007/s00709-022-01741-6 35146572

[B8] Aledwany A. Basal W. Al-Senosy N. Issa A. (2018). Assessment of genotoxicity of potassium nitrate and sodium benzoate in Drosophila melanogaster using smart and comet assays. Egypt Acad. J. Biol. Sci. C Physiol. Mol. Biol. 10, 83–97. 10.21608/eajbsc.2018.22715

[B9] Ali M. Y. Hassan G. M. Hassan A. M. S. Mohamed Z. A. Ramadan M. F. (2020). *In vivo* genotoxicity assessment of sunset yellow and sodium benzoate in female rats. Drug Chem. Toxicol. 43, 504–513. 10.1080/01480545.2018.1510416 30208729

[B10] Ali H. H. Alharbi S. F. Iskandar R. A. Mira G. B. Yanogue A. S. Alboualy E. A. (2024). Perception and use of herbal medicine in general practice patients: a cross-sectional study in Saudi Arabia. Cureus 16, e56806. 10.7759/cureus.56806 38654786 PMC11036024

[B11] Almutairi K. De Santis J. (2024). Prophetic medicine in the context of Middle Eastern culture: a concept analysis. Res. Theory Nurs. Pract. 39, 55–78. 10.1891/RTNP-2023-0158 39152043

[B12] Alrekabi D. G. Hamad M. N. (2018). Phytochemical investigation of Sonchus oleraceus (Family:Asteraceae) cultivated in Iraq, isolation and identification of quercetin and apigenin. J. Pharm. Sci. Res. 10, 2242–2248.

[B13] Althubyani M. A. Alrefaei A. F. (2024). Protective and therapeutic effects of medicinal plants against food additive-induced toxicity. Pak. J. Biol. Sci. 27, 439–446. 10.3923/pjbs.2024.439.446 39415552

[B14] Alzandi A. A. Taher E. A. Al-Sagheer N. A. Al-Khulaidi A. W. Azizi M. Naguib D. M. (2021). Phytochemical components, antioxidant and anticancer activity of 18 major medicinal plants in albaha region, Saudi Arabia. Biocatal. Agric. Biotechnol. 34, 102020. 10.1016/j.bcab.2021.102020

[B15] Ananthi R. Chandra N. Santhiya S. T. Ramesh A. (2010). Genotoxic and antigenotoxic effects of Hemidesmus indicus R. Br. Root extract in cultured lymphocytes. J. Ethnopharmacol. 127, 558–560. 10.1016/j.jep.2009.10.034 19896526

[B16] Atienzar F. A. Conradi M. Evenden A. J. Jha A. N. Depledge M. H. (1999). Qualitative assessment of genotoxicity using random amplified polymorphic DNA: comparison of genomic template stability with key fitness parameters in Daphnia magna exposed to benzo[a]pyrene. Environ. Toxicol. Chem. 18, 2275–2282. 10.1002/etc.5620181023 29857629

[B17] Awadh Ali N. A. Al Sokari S. S. Gushash A. Anwar S. Al-Karani K. Al-Khulaidi A. (2017). Ethnopharmacological survey of medicinal plants in albaha region, Saudi Arabia. Pharmacogn. Res. 9, 401–407. 10.4103/pr.pr_11_17 29263636 PMC5717795

[B18] Barman M. Ray S. (2023). Cytogenotoxic effects of 3-Epicaryoptin in Allium cepa L. root apical meristem cells. Protoplasma 260, 1163–1177. 10.1007/s00709-023-01838-6 36735079

[B19] Benjamini Y. Hochberg Y. (1995). Controlling the false discovery rate: a practical and powerful approach to multiple testing. J. R. Stat. Soc. Ser. B Methodol. 57, 289–300. 10.1111/j.2517-6161.1995.tb02031.x

[B20] Bernardes P. M. Andrade-Vieira L. F. Aragão F. B. Ferreira A. Ferreira M. F. S. (2015). Toxicity of difenoconazole and tebuconazole in Allium cepa. Water Air Soil Pollut. 226, 1–11. 10.1007/s11270-015-2462-y

[B21] Boukandou Mounanga M. Mewono L. Aboughe Angone S. (2015). Toxicity studies of medicinal plants used in Sub-Saharan Africa. J. Ethnopharmacol. 174, 618–627. 10.1016/j.jep.2015.06.005 26087230

[B22] Çelik T. A. Aslantürk Ö. S. (2010). Evaluation of cytotoxicity and genotoxicity of Inula viscosa leaf extracts with allium test. J. Biomed. Biotechnol. 2010, 189252. 10.1155/2010/189252 20617136 PMC2896651

[B23] Chen L. Fan X. Lin X. Qian L. Zengin G. Delmas D. (2020). Phenolic extract from Sonchus oleraceus L. protects diabetes-related liver injury in rats through TLR4/NF-ΚB signaling pathway. eFood 1, 77–84. 10.2991/efood.k.191018.002

[B24] Chen G.-H. Song C.-C. Pantopoulos K. Wei X.-L. Zheng H. Luo Z. (2022a). Mitochondrial oxidative stress mediated Fe-Induced ferroptosis *via* the NRF2-ARE pathway. Free Radic. Biol. Med. 180, 95–107. 10.1016/j.freeradbiomed.2022.01.012 35045311

[B25] Chen X. Hongyan L. Bing Z. Deng Z. (2022b). The synergistic and antagonistic antioxidant interactions of dietary phytochemical combinations. Crit. Rev. Food Sci. Nutr. 62, 5658–5677. 10.1080/10408398.2021.1888693 33612011

[B26] Chukwujekwu J. C. Van Staden J. (2014). Cytotoxic and genotoxic effects of water extract of Distephanus angulifolius on Allium cepa linn. South Afr. J. Bot. 92, 147–150. 10.1016/j.sajb.2014.03.001

[B27] Clifford H. T. Clayton W. D. Renvoize S. A. (2022). Genera graminum. Grasses of the world. Kew Bull. 45, 208. 10.2307/4114451

[B28] Couto V. M. Vilela F. C. Dias D. F. Dos Santos M. H. Soncini R. Nascimento C. G. O. (2011). Antinociceptive effect of extract of Emilia sonchifolia in mice. J. Ethnopharmacol. 134, 348–353. 10.1016/j.jep.2010.12.028 21185930

[B29] Daradka H. Aljohani H. Alotaibi M. Khabour O. Eskandrani A. Alsharif S. (2021). Evaluating the effects of commiphora molmol (myrrh) against oxidative DNA damage in human lymphocytes. Int. J. Pharm. Sci. Res. 12, 3143–3149. 10.13040/IJPSR.0975-8232.12(6).3143-49

[B30] Dey K. K. Kamila S. Das T. Chattopadhyay A. (2024). Lead induced genotoxicity and hepatotoxicity in zebrafish (Danio rerio) at environmentally relevant concentration: nrf2-keap1 regulated stress response and expression of biomarker genes. Environ. Toxicol. Pharmacol. 107, 104396. 10.1016/j.etap.2024.104396 38395243

[B31] Dragoeva A. P. Koleva V. P. Nanova Z. D. Georgiev B. P. (2015). Allelopathic effects of adonis vernalis L.: root growth inhibition and cytogenetic alterations. J. Agric. Chem. Environ. 04 (04), 48–55. 10.4236/jacen.2015.42005

[B32] Dunford E. K. Miles D. R. Popkin B. (2023). Food additives in ultra-processed packaged foods: an examination of US household grocery Store purchases. J. Acad. Nutr. Diet. 123, 889–901. 10.1016/j.jand.2022.11.007 36931919 PMC10200736

[B33] Ekor M. (2014). The growing use of herbal medicines: issues relating to adverse reactions and challenges in monitoring safety. Front. Pharmacol. 4, 177. 10.3389/fphar.2013.00177 24454289 PMC3887317

[B34] El Gendy A. E. N. G. Mohamed N. A. Sarker T. C. Hassan E. M. Garaa A. H. Elshamy A. I. (2024). Chemical composition, antioxidant, and cytotoxic activity of essential oils in the above-ground parts of Sonchus oleraceus L. Plants 13, 1712. 10.3390/plants13121712 38931144 PMC11207314

[B35] El Ghazali G. Al-Khalifa K. Saleem G. Abdallah E. (2010). Traditional medicinal plants Indigenous to Al-Rass province, Saudi Arabia. J. Med. Plants Res. 4, 2680–2683. 10.5897/JMPR09.556

[B36] El-Saadony M. T. Saad A. M. Mohammed D. M. Korma S. A. Alshahrani M. Y. Ahmed A. E. (2025). Medicinal plants: bioactive compounds, biological activities, combating multidrug-resistant microorganisms, and human health benefits - a comprehensive review. Front. Immunol. 16, 1491777. 10.3389/fimmu.2025.1491777 40375989 PMC12079674

[B37] Elkhayat E. (2009). Cytotoxic and antibacterial constituents from the roots of Sonchus oleraceus L. growing in Egypt. Pharmacogn. Mag. - Pharmacogn. Mag. 5, 324. 10.4103/0973-1296.58154

[B38] Enan M. R. (2007). Assessment of genotoxic activity of para-nitrophenol in higher plant using arbitrarily primed-polymerase chain reaction (AP-PCR). Am. J. Biotechnol. Biochem. 3, 103–109. 10.3844/ajbbsp.2007.103.109

[B39] Ene E. E. Osuala C. L. (1990). The mutagenic potentials of water extracts of Borreria filiformis (hiern) hatch and dalz. and vince rosea linn. Niger. J. Bot. 3, 35–40.

[B40] Eroz Poyraz I. Poyraz I. Kıyan H. Öztürk N. Erken S. Gülbağ F. (2018). Detection of the genotoxicity of gentiana L. extracts by using RAPD-PCR and ISSR-PCR techniques. Indian J. Pharm. Educ. Res. 42, 133–139. 10.5530/ijper.52.4s.89

[B41] Ferguson L. R. (1994). Antimutagens as cancer chemopreventive agents in the diet. Mutat. Research/Fundamental Mol. Mech. Mutagen. 307, 395–410. 10.1016/0027-5107(94)90313-1 7513820

[B42] Ferguson L. R. (2001). Role of plant polyphenols in genomic stability. Mutat. Research/Fundamental Mol. Mech. Mutagen. 475, 89–111. 10.1016/S0027-5107(01)00073-2 11295156

[B43] Ferguson L. R. Philpott M. Karunasinghe N. (2004). Dietary cancer and prevention using antimutagens. Toxicology 198, 147–159. 10.1016/j.tox.2004.01.035 15138038

[B44] Gaur H. Purushothaman S. Pullaguri N. Bhargava Y. Bhargava A. (2018). Sodium benzoate induced developmental defects, oxidative stress and anxiety-like behaviour in zebrafish larva. Biochem. Biophys. Res. Commun. 502, 364–369. 10.1016/j.bbrc.2018.05.171 29842881

[B45] Geirid Allium Test for Screening Chemicals (1997). Evaluation of cytological parameters.

[B46] Guo X. Wang X. Su W. Zhang G. Zhou R. (2011). DNA barcodes for discriminating the medicinal plant Scutellaria baicalensis (lamiaceae) and its adulterants. Biol. Pharm. Bull. 34, 1198–1203. 10.1248/bpb.34.1198 21804206

[B47] Haglund L. (2022). Optimised PCR protocol for ten microsatellite primers (SSRs) in fragaria vesca: facilitating future work analysing genetic diversity and developing efficient conservation strategies.

[B48] Hayakawa F. Kimura T. Hoshino N. Ando T. (1999). DNA cleavage activities of (-)-epigallocatechin, (-)-epicatechin, (+)-catechin, and (-)-epigallocatechin gallate with various kinds of metal ions. Biosci. Biotechnol. Biochem. 63, 1654–1656. 10.1271/bbb.63.1654 10610127

[B49] Hilal B. Khan M. M. Fariduddin Q. (2024). Recent advancements in deciphering the therapeutic properties of plant secondary metabolites: phenolics, terpenes, and alkaloids. Plant Physiology Biochem. 211, 108674. 10.1016/j.plaphy.2024.108674 38705044

[B50] Hussain J. Muhammad Z. Ullah R. Khan F. Khan I. Khan N. (2010). Evaluation of the chemical composition of sonchus eruca and Sonchus asper. J. Am. Sci. 6, 231–235.

[B51] Jimoh F. Adedapo A. Afolayan A. (2011). Comparison of the nutritive value, antioxidant and antibacterial activities of Sonchus asper and Sonchus oleraceus. Rec. Nat. Prod. 51, 29–42.

[B52] Juan C. Perez de la lastra J. Plou F. Lebeña E. (2021). The chemistry of reactive oxygen species (ROS) revisited: outlining their role in biological macromolecules (DNA, lipids and proteins) and induced pathologies. Int. J. Mol. Sci. 22, 4642. 10.3390/ijms22094642 33924958 PMC8125527

[B53] Kameswari D. Ezhil V. Perumal V. Manju M. Meganathan M. Madhanagopal K. (2023). Effect of Azadirachta indica against sodium benzoate induced hepatorenal toxicity in wistar Rats- an experimental interventional study. J. Clin. Diagnostic Res. 17, 12–16. 10.7860/jcdr/2023/59151.17866

[B54] Kaurinovic B. Vastag D. (2019). “Flavonoids and phenolic acids as potential natural antioxidants,” in Antioxidants. Editor Shalaby E. (Rijeka: IntechOpen).

[B55] Khanna N. Sharma S. (2013). Allium cepa root chromosomal aberration assay: a review. Indian J. Pharm. Biol. Res. 1, 105–119. 10.30750/ijpbr.1.3.15

[B56] Khare C. P. (2007). Launaea pinnatifida cass. Indian Med. Plants, 1. 10.1007/978-0-387-70638-2_887

[B57] Khoshnoud M. J. Siavashpour A. Bakhshizadeh M. Rashedinia M. (2018). Effects of sodium benzoate, a commonly used food preservative, on learning, memory, and oxidative stress in brain of mice. J. Biochem. Mol. Toxicol. 32, e22022. 10.1002/jbt.22022 29243862

[B58] Kumar G. Pandey A. (2015). Genotoxic and mito-depressive effects of food preservatives on root meristems of barley (Hordeum vulgare L.). Chromosom. Bot. 10, 51–60. 10.3199/iscb.10.51

[B59] Kusumaningrum H. P. Lunggani A. T. Nurhakim M. A. (2012). Chromosomes and mitotic cell division phase in onion roots after 24 hours acetoorcein soaking time. Bioma Berk. Ilm. Biol. 14, 46. 10.14710/bioma.14.2.46-48

[B60] Leme D. M. Marin-Morales M. A. (2009). Allium cepa test in environmental monitoring: a review on its application. Mutat. Research/Reviews Mutat. Res. 682, 71–81. 10.1016/j.mrrev.2009.06.002 19577002

[B61] Lennerz B. S. Vafai S. B. Delaney N. F. Clish C. B. Deik A. A. Pierce K. A. (2015). Effects of sodium benzoate, a widely used food preservative, on glucose homeostasis and metabolic profiles in humans. Mol. Genet. Metab. 114, 73–79. 10.1016/j.ymgme.2014.11.010 25497115 PMC4289147

[B62] Lenth R. V. Emmeans S. H. (2025). Estimated marginal means, Aka least-squares means (R package version 1.8.9). Available online at: https://CRAN.R-project.org/package=emmeans (accessed on September 1, 2025).

[B63] Lestari B. Novitasari D. Putri H. Haryanti S. Ediati S. Meiyanto E. (2017). Evaluation of the genotoxicity of three food additives using CHO-K1 cells under *in vitro* micronucleus flow cytometry assay. Indonesian J. Cancer Chemoprevention 8, 74. 10.14499/indonesianjcanchemoprev8iss2pp74-80

[B64] Li L. Y. Guan Y.D. Chen X. S. Yang J. M. Cheng Y. (2021). DNA repair pathways in cancer therapy and resistance. Front. Pharmacol. 11, 629266. 10.3389/fphar.2020.629266 33628188 PMC7898236

[B65] Lubini G. Fachinetto J. M. Laughinghouse H. D. Paranhos J. T. Silva A. C. F. Tedesco S. B. (2008). Extracts affecting mitotic division in root-tip meristematic cells. Biol. Bratisl. 63, 647–651. 10.2478/s11756-008-0108-x

[B66] Marques R. C. P. de Medeiros S. R. B. Dias C. da S. Barbosa-Filho J. M. Agnez-Lima L. F. (2003). Evaluation of the mutagenic potential of yangambin and of the hydroalcoholic extract of Ocotea duckei by the ames test. Mutat. Res. 536, 117–120. 10.1016/s1383-5718(03)00040-8 12694751

[B67] Marri P. Neelamraju S. Siddiq E. A. (2002). Inter simple sequence repeat (ISSR) polymorphism and its application in plant breeding. Euphytica 128, 9–17. 10.1023/A:1020691618797

[B68] Medeiros Vinci R. Jacxsens L. Van Loco J. Matsiko E. Lachat C. de Schaetzen T. (2012). Assessment of human exposure to benzene through foods from the Belgian market. Chemosphere 88, 1001–1007. 10.1016/j.chemosphere.2012.03.044 22483726

[B69] Melo-Reis P. R. Bezerra L. S. A. Vale M. A. A. B. Canhête R. F. R. Chen-Chen L. (2011). Assessment of the mutagenic and antimutagenic activity of synadenium umbellatum pax latex by micronucleus test in mice. Braz J. Biol. 71, 169–174. 10.1590/s1519-69842011000100024 21437414

[B70] Mohammed J. S. Mustapha Y. Him M. A. Danladi Z. N. (2023). Assessment of cytogenotoxicity of plastic industrial effluent using Allium cepa root tip cells. Int. J. Cell Biol. 2023, 5161017. 10.1155/2023/5161017 37881210 PMC10597712

[B71] Montera V. dos S. P. Martins A. P. B. Borges C. A. Canella D. S. (2021). Distribution and patterns of use of food additives in foods and beverages available in Brazilian supermarkets. Food Funct. 12, 7699–7708. 10.1039/D1FO00429H 34282819

[B72] Nicuta D. Grosu L. Patriciu O.-I. Voicu R.-E. Alexa I.-C. (2025). The Allium cepa model: a review of its application as a cytogenetic tool for evaluating the biosafety potential of plant extracts. Methods Protoc. 8, 88. 10.3390/mps8040088 40863738 PMC12388284

[B73] Nogueira M. E. I. Passoni M. H. Biso F. I. Longo M. do C. Cardoso C. R. P. Santos L.C. dos (2006). Investigation of genotoxic and antigenotoxic activities of Melampodium divaricatum in Salmonella typhimurium. Toxicol. Vitro 20, 361–366. 10.1016/j.tiv.2005.08.012 16182509

[B74] Oladele J. O. Oladele O. T. Ademiluyi A. O. Oyeleke O. M. Awosanya O. O. Oyewole O. I. (2020). A systematic review on COVID-19 pandemic with special emphasis on curative potentials of Nigeria based medicinal plants. Clin. Phytoscience 6, e04897. 10.1016/j.heliyon.2020.e04897 PMC7480258 32929412

[B75] Oulahal N. Degraeve P. (2022). Phenolic-rich plant extracts with antimicrobial activity: an alternative to food preservatives and biocides? Front. Microbiol. 12, 753518. 10.3389/fmicb.2021.753518 35058892 PMC8764166

[B76] Pereira-Wilson C. Ramos A. Lima C. Lima C. (2011). “DNA damage protection and induction of repair by dietary phytochemicals and cancer prevention: what do we know?,” in Selected topics in DNA repair. Editor Chen C. (Rijeka: IntechOpen).

[B77] Pérez-Carreón J. I. Cruz-Jiménez G. Licea-Vega J. A. Arce Popoca E. Fattel Fazenda S. Villa-Treviño S. (2002). Genotoxic and anti-genotoxic properties of Calendula officinalis extracts in rat liver cell cultures treated with diethylnitrosamine. Toxicol. Vitro 16, 253–258. 10.1016/S0887-2333(02)00005-X 12020598

[B78] Ping K. Y. Darah I. Yusuf U. K. Yeng C. Sasidharan S. (2012). Genotoxicity of euphorbia hirta: an Allium cepa assay. Molecules 17, 7782–7791. 10.3390/molecules17077782 22735780 PMC6269077

[B79] Pongsavee M. (2015). Effect of sodium benzoate preservative on micronucleus induction, chromosome break, and Ala40Thr superoxide dismutase gene mutation in lymphocytes. Biomed. Res. Int. 2015, 103512. 10.1155/2015/103512 25785261 PMC4346689

[B80] Poyraz I. (2021). An investigation of the genotoxic and cytotoxic effects of myclobutanil fungicide on plants. Plant Prot. Sci. 58, 57–64. 10.17221/6/2021-PPS

[B81] Qari S. H. (2016). Cytotoxic and genotoxic assessment of Citrullus colocynthis. Int. J. Sci. Res. Rev. (IJSRR) 5, 20–39.

[B82] Qari S. H. (2017). DNA flow cytometric and cytogenetic studies on Allium cepa L. Root tips treated with Trigonella hamosa L. and/or sodium benzoate. arXiv 24, 233–248.

[B83] R Core Team R (2025). A language and environment for statistical computing. Vienna, Austria: R Foundation for Statistical Computing. Available online at: https://www.R-project.org/ (accessed on September 1, 2025).

[B84] Rasool N. Omer O. Javeed A. Nawaz M. Rasheed M. Imran M. (2023). Phytochemical properties and *in-vitro* cytotoxicity, genotoxicity and mutagenicity assessment of ethanolic and aqueous extracts of Argyrolobium roseum (camb.). Int. J. Food Prop. 26, 1457–1469. 10.1080/10942912.2023.2219859

[B85] Regner G. G. Gianesini J. Von Borowski R. G. Silveira F. Semedo J. G. Ferraz A. de B. F. (2011). Toxicological evaluation of Pterocaulon polystachyum extract: a medicinal plant with antifungal activity. Environ. Toxicol. Pharmacol. 31, 242–249. 10.1016/j.etap.2010.11.003 21787691

[B86] Rybczyńska-Tkaczyk K. Skóra B. Szychowski K. A. (2023). Toxicity of bisphenol A (BPA) and its derivatives in divers biological models with the assessment of molecular mechanisms of toxicity. Environ. Sci. Pollut. Res. 30, 75126–75140. 10.1007/s11356-023-27747-y 37213006 PMC10293331

[B87] Saatci C. Erdem Y. Bayramov R. Akalın H. Tascioglu N. Ozkul Y. (2016). Effect of sodium benzoate on DNA breakage, micronucleus formation and mitotic index in peripheral blood of pregnant rats and their newborns. Biotechnol. and Biotechnol. Equip. 30, 1179–1183. 10.1080/13102818.2016.1224979

[B88] Salim N. S. Abdel-Alim M. Said H. E. M. Foda M. F. (2023). Phenolic profiles, antihyperglycemic, anti-diabetic, and antioxidant properties of Egyptian Sonchus oleraceus leaves extract: an *in vivo* study. Molecules 28, 6389. 10.3390/molecules28176389 37687218 PMC10489745

[B89] Salas-Coronado R. Santos-Sánchez N. F. Hernández-Carlos B. Villanueva-Cañongo C. (2019). “Antioxidant compounds and their antioxidant mechanism,” in Antioxidants. Editor Shalaby E. (Rijeka: IntechOpen).

[B90] Sánchez-Aguirre O. A. Sánchez-Medina A. Juárez-Aguilar E. Barreda-Castillo J. M. Cano-Asseleih L. M. (2024). Sonchus oleraceus L.: Ethnomedical, phytochemical and pharmacological aspects. Naunyn Schmiedeb. Arch. Pharmacol. 397 (7), 4555–4578. 10.1007/s00210-024-02966-3 38305867

[B91] Sarhan M. A. A. (2010). Cytotoxicity and genotoxicity potential of thiocyclam in root-tip cells of Allium cepa. arXiv 6, 601–608.

[B92] Saxena P. N. Gupta S. K. Murthy R. C. (2010). Carbofuran induced cytogenetic effects in root meristem cells of Allium cepa and allium sativum: a spectroscopic approach for chromosome damage. Pestic. Biochem. Physiol. 96, 93–100. 10.1016/j.pestbp.2009.09.006

[B93] Sharif A. Akhtar M. F. Akhtar B. Saleem A. Manan M. Shabbir M. (2017). Genotoxic and cytotoxic potential of whole plant extracts of Kalanchoe laciniata by ames and MTT assay. EXCLI J. 16, 593–601. 10.17179/excli2016-748 28694760 PMC5491922

[B94] Sher H. Aldosari A. (2012). Overview on the ecological and geographical appraisal of important medicinal and aromatic plants: an endangered component in the flora of Saudi Arabia. Sci. Res. Essays 7, 1639–1646. 10.5897/sre12.183

[B95] Shields H. J. Traa A. Van Raamsdonk J. M. (2021). Beneficial and detrimental effects of reactive oxygen species on lifespan: a comprehensive review of comparative and experimental studies. Front. Cell Dev. Biol. 9, 628157. 10.3389/fcell.2021.628157 33644065 PMC7905231

[B96] Shin I. S. Seo C. S. Ha H. K. Lee M. Y. Huang D. S. Huh J. I. (2011). Genotoxicity assessment of pyungwi-san (PWS), a traditional herbal prescription. J. Ethnopharmacol. 133, 696–703. 10.1016/j.jep.2010.10.050 21040766

[B97] Silva C. R. Monteiro M. R. Rocha H. M. Ribeiro A. F. Caldeira-de-Araujo A. Leitão A. C. (2008). Assessment of antimutagenic and genotoxic potential of senna (Cassia angustifolia vahl.) aqueous extract using *in vitro* assays. Toxicol. Vitro 22, 212–218. 10.1016/j.tiv.2007.07.008 17826029

[B98] Sponchiado G. Adam M. L. Silva C. D. Silva Soley B. de Mello-Sampayo C. Cabrini D. A. (2016). Quantitative genotoxicity assays for analysis of medicinal plants: a systematic review. J. Ethnopharmacol. 178, 289–296. 10.1016/j.jep.2015.10.026 26680588

[B99] Sreenivasan A. Rekha G S. A. Prakash V. R P. G. (2023). Pharmaceutical sciences a review on the use of sodium benzoate as a preservative in carbonated softdrinks. arXiv 10, 129–135. 10.5281/zenodo.8124434

[B100] Steele V. E. Kelloff G. J. (2005). Development of cancer chemopreventive drugs based on mechanistic approaches. Mutat. Research/Fundamental Mol. Mech. Mutagen. 591, 16–23. 10.1016/j.mrfmmm.2005.04.018 16083917

[B101] Sudha G. Ramesh P. Akila C. S. Talla S. Chari B. Riazunnisa K. (2018). Genetic diversity analysis of selected onion (Allium cepa L.) germplasm using specific RAPD and ISSR polymorphism markers. Biocatal. Agric. Biotechnol. 17. 10.1016/j.bcab.2018.11.007

[B102] Teugwa C. M. Mejiato P. C. Zofou D. Tchinda B. T. Boyom F. F. (2013). Antioxidant and antidiabetic profiles of two African medicinal plants: picralima nitida (apocynaceae) and Sonchus oleraceus (asteraceae). BMC Complement. Altern. Med. 13, 175. 10.1186/1472-6882-13-175 23855679 PMC3718716

[B103] Tounekti T. Mahdhi M. Khemira H. (2019). Ethnobotanical study of Indigenous medicinal plants of jazan region, Saudi Arabia. Evidence-Based Complementary Altern. Med. 2019, 3190670. 10.1155/2019/3190670 31275409 PMC6582903

[B104] Tsay H.-J. Wang Y.-H. Chen W.-L. Huang M.-Y. Chen Y.-H. (2007). Treatment with sodium benzoate leads to malformation of zebrafish larvae. Neurotoxicol Teratol. 29, 562–569. 10.1016/j.ntt.2007.05.001 17644306

[B105] Vecchia C. A. D. Locateli G. Serpa P. Z. Bianchin Gomes D. Ernetti J. Miorando D. (2022). Sonchus oleraceus L. promotes gastroprotection in rodents *via* antioxidant, anti-inflammatory, and antisecretory activities. Evidence-Based Complementary Altern. Med. 2022, 7413231. 10.1155/2022/7413231 36051492 PMC9427263

[B106] Verma S. Singh S. P. (2008). Current and future status of herbal medicines. Vet. World 1, 347–350. 10.5455/vetworld.2008.347-350

[B107] Verschaeve L. Van Staden J. (2008). Mutagenic and antimutagenic properties of extracts from South African traditional medicinal plants. J. Ethnopharmacol. 119, 575–587. 10.1016/j.jep.2008.06.007 18602977

[B108] Vujčić Bok V. Gerić M. Gajski G. Gagić S. Domijan A. M. (2023). Phytotoxicity of bisphenol A to Allium cepa root cells is mediated through growth hormone gibberellic acid and reactive oxygen species. Molecules 28, 2046. 10.3390/molecules28052046 36903292 PMC10004651

[B109] Xia D. Yu X.-F. Zhu Z.-Y. Zou Z.-D. (2011). Antioxidant and antibacterial activity of six edible wild plants (sonchus spp.) in China. Nat. Prod. Res. 25, 1893–1901. 10.1080/14786419.2010.534093 21793765

[B110] Yamanaka N. Oda O. Nagao S. (1997). Green tea catechins such as (−)-Epicatechin and (−)-Epigallocatechin accelerate Cu2+-Induced low density lipoprotein oxidation in propagation phase. FEBS Lett. 401, 230–234. 10.1016/S0014-5793(96)01455-X 9013893

[B111] Yassien E. E. Mohamed A. M. S. Mahmoud M. E. Zaki A. M. (2022). Sodium benzoate induced toxicities in albino Male rats: mitigating effects of ficus carica and Cymbopogon citratus leave extract. Environ. Sci. Pollut. Res. Int. 29, 90567–90579. 10.1007/s11356-022-22020-0 35871196

[B112] Yetuk G. Pandir D. Bas H. (2014). Protective role of catechin and quercetin in sodium benzoate-induced lipid peroxidation and the antioxidant system in human erythrocytes *in vitro* . Sci. World J. 2014, 874824. 10.1155/2014/874824 24693251 PMC3943299

[B113] Yılmaz S. Ünal F. Yüzbaşıoğlu D. (2009). The *in vitro* genotoxicity of benzoic acid in human peripheral blood lymphocytes. Cytotechnology 60, 55. 10.1007/s10616-009-9214-z 19642007 PMC2780543

[B114] Yin J. Kwon G.-J. Wang M.-H. (2007). The antioxidant and cytotoxic activities of Sonchus oleraceus L. extracts. Nutr. Res. Pract. 1, 189–194. 10.4162/nrp.2007.1.3.189 20368937 PMC2849021

[B115] Zar J. (2013). Biostatistical analysis. Deutschland: Pearson.

[B116] Zeiger E. (2003). Illusions of safety: antimutagens can be mutagens, and anticarcinogens can be carcinogens. Mutat. Res. 543, 191–194. 10.1016/S1383-5742(02)00111-4 12787811

[B117] Zengin N. Yüzbaşıoğlu D. Ünal F. Yılmaz S. Aksoy H. (2011). The evaluation of the genotoxicity of two food preservatives: sodium benzoate and potassium benzoate. Food Chem. Toxicol. 49, 763–769. 10.1016/j.fct.2010.11.040 21130826

[B118] Zhang G. Ma Y. (2013). Spectroscopic studies on the interaction of sodium benzoate, a food preservative, with calf thymus DNA. Food Chem. 141, 41–47. 10.1016/j.foodchem.2013.02.122 23768324

[B119] Zietkiewicz E. Rafalski A. Labuda D. (1994). Genome fingerprinting by simple sequence repeat (SSR)-anchored polymerase chain reaction amplification. Genomics 20, 176–183. 10.1006/geno.1994.1151 8020964

[B120] Zuur A. F. (2010). Mixed effects models and extensions in ecology with R. Springer Science and Business Media.

