# Supplementary material for: Biological analysis of Sonchus oleraceus (Linn) extract and its effect on mitigating sodium benzoate-induced cytotoxicity and genotoxicity
Source: Front Toxicol. 2025 Nov 13;7:1674822. doi: 10.3389/ftox.2025.1674822 (PMC12658989; doi:10.3389/ftox.2025.1674822)
Supplement: Supplementary file 1 [file Supplementaryfile1.docx]

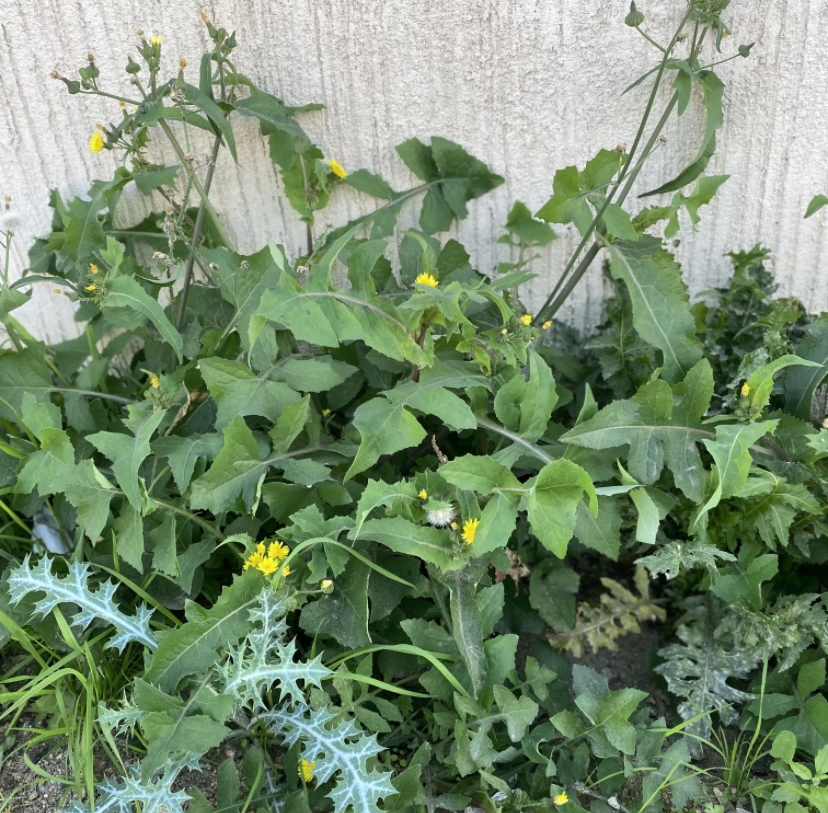


**Figure S1.** The Picture of *Sonchus oleraceus (Linn).*


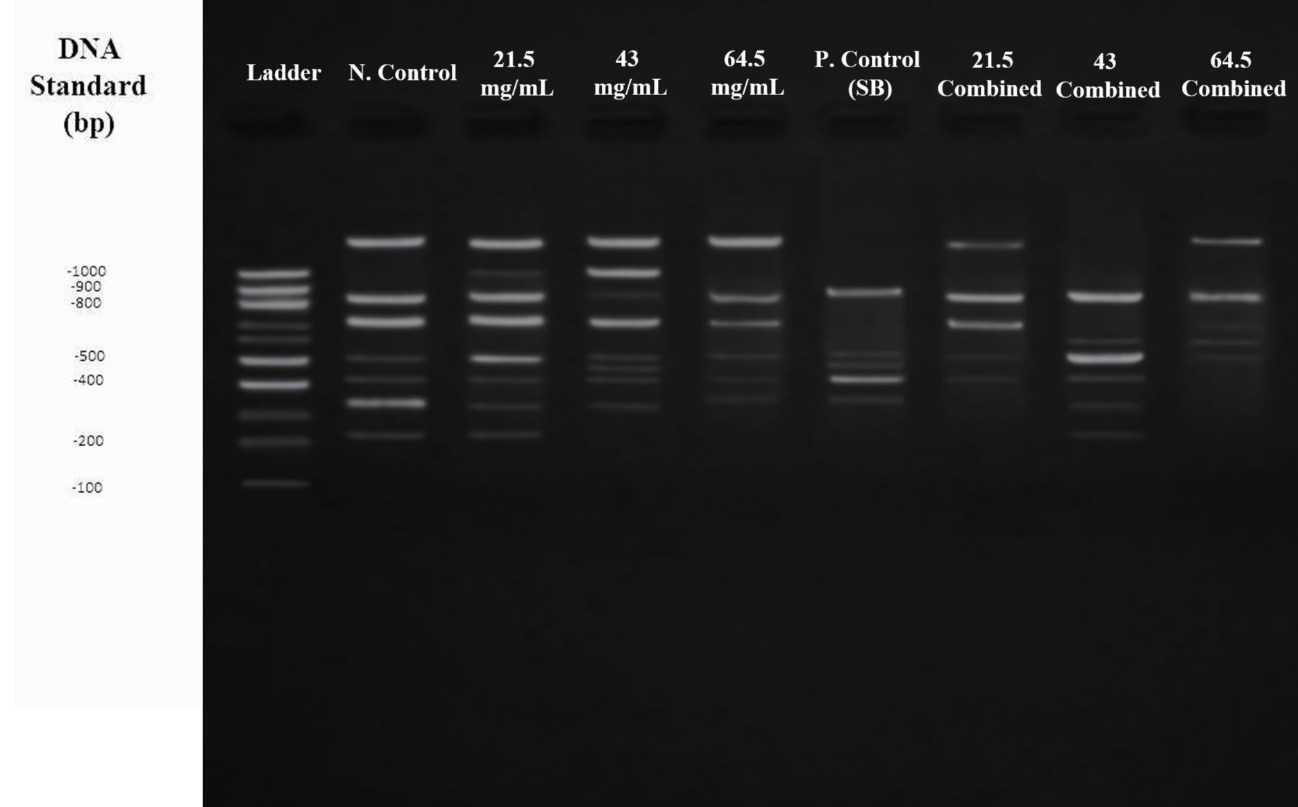


**
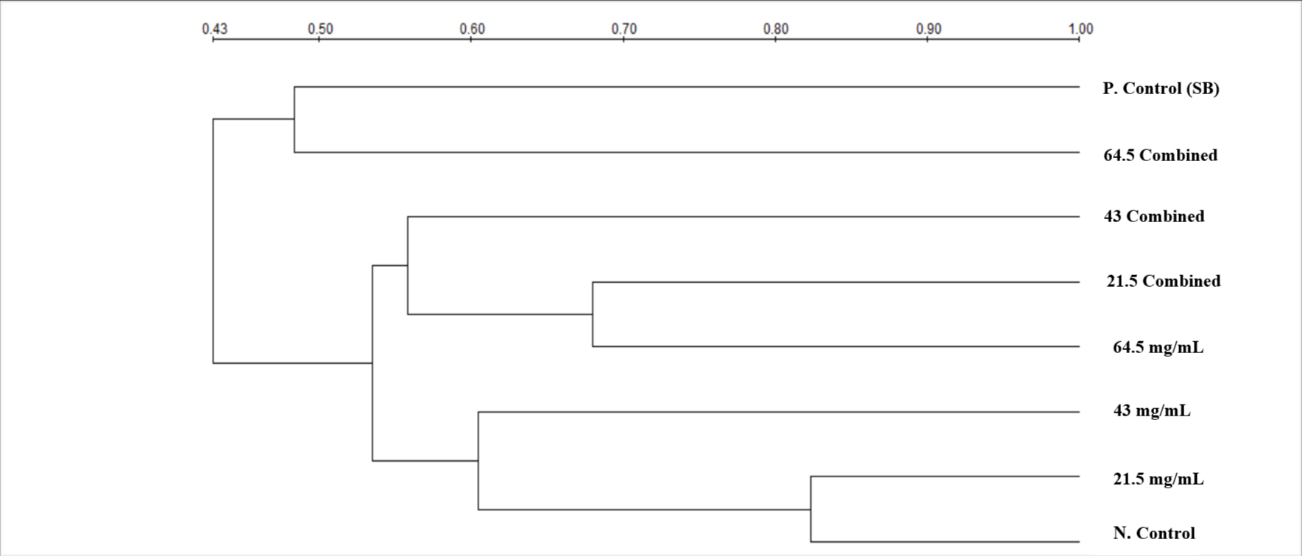
Figure S2.** PCR products of ISSR-HB12 marker amplified with DNA treated with different concentrations of *S. oleraceus* extract as a single and combined treatment compared to negative control (dH₂O) for 48 hr.

**Figure S3.** The phylogenetic tree by UPGMA of ISSR-HB12 marker for different concentrations and treatments of *S. oleraceus* extract as a single and combined treatment compared to the negative control (dH₂O).


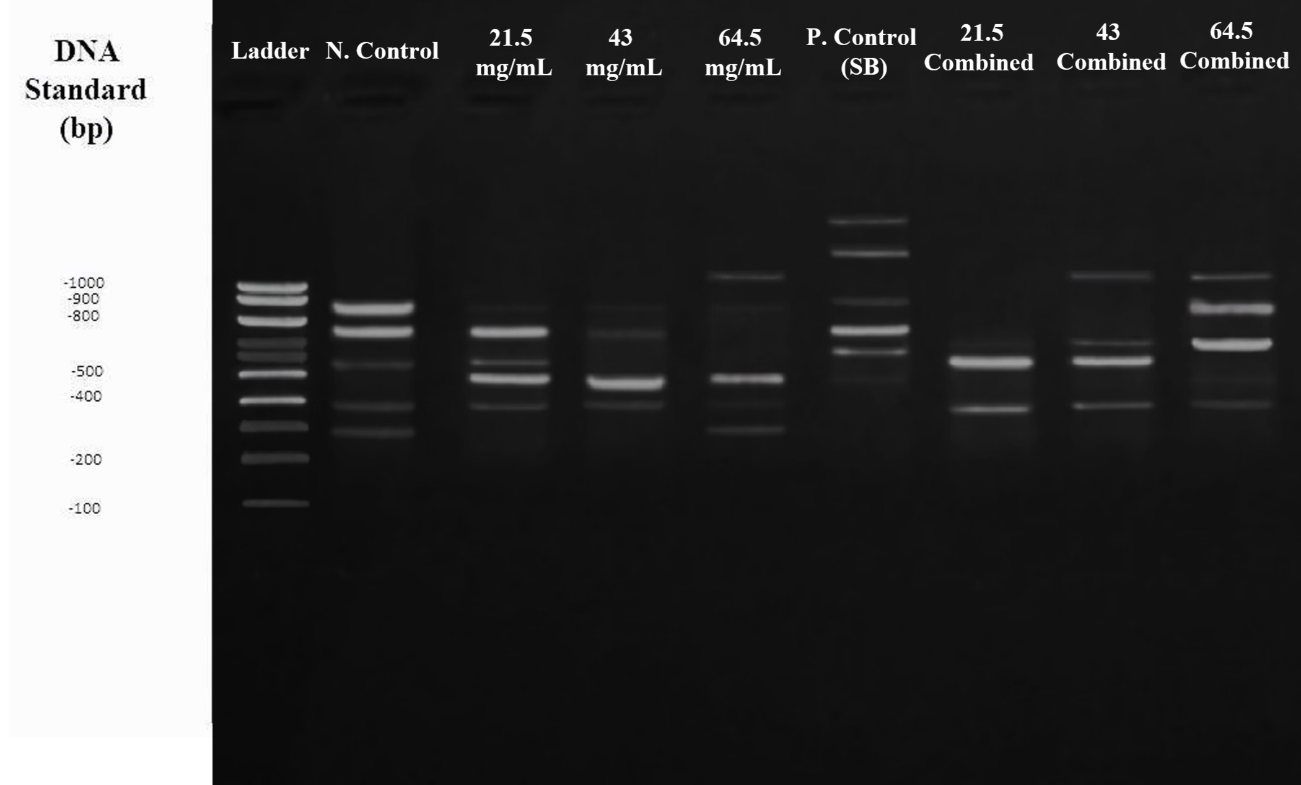


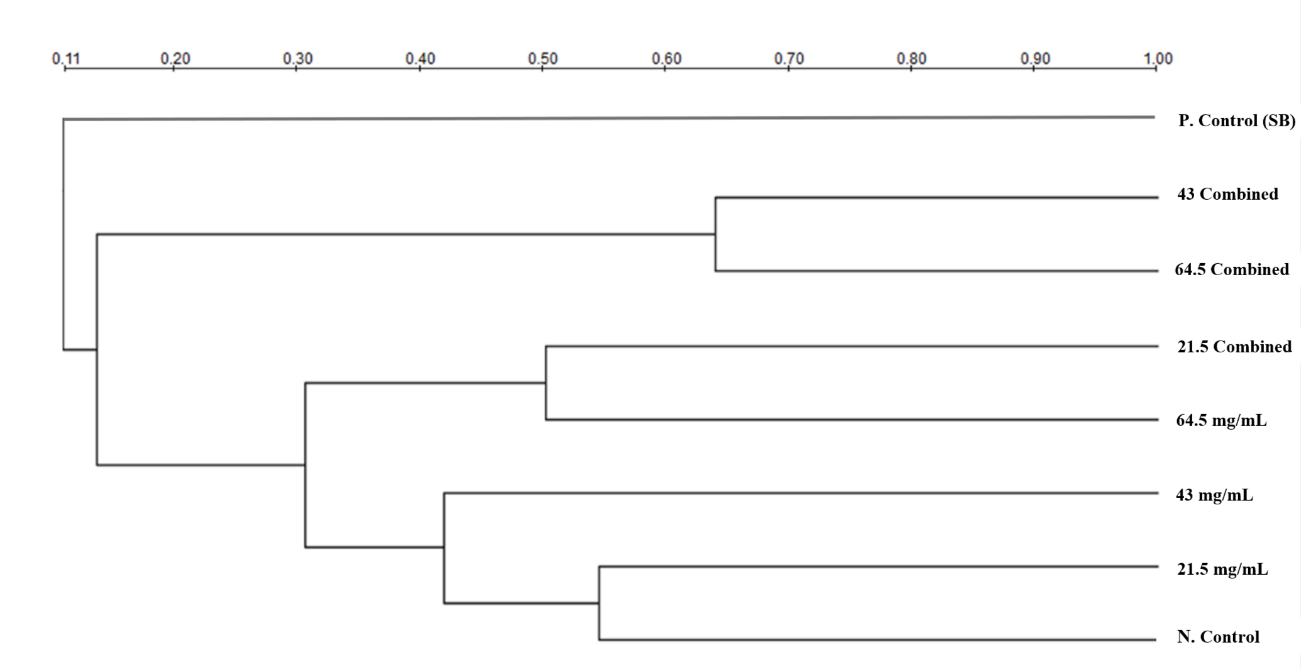
 **Figure S4.** PCR products of ISSR-418 marker amplified with DNA treated with different concentrations of *S. oleraceus* extract as a single and combined treatment compared to negative control (dH₂O) for 48 hr.

**Figure S5.** The phylogenetic by UPGMA of ISSR-418 marker for different concentrations and treatments of *S. oleraceus* extract as a single and combined treatment compared to the negative control (dH₂O).


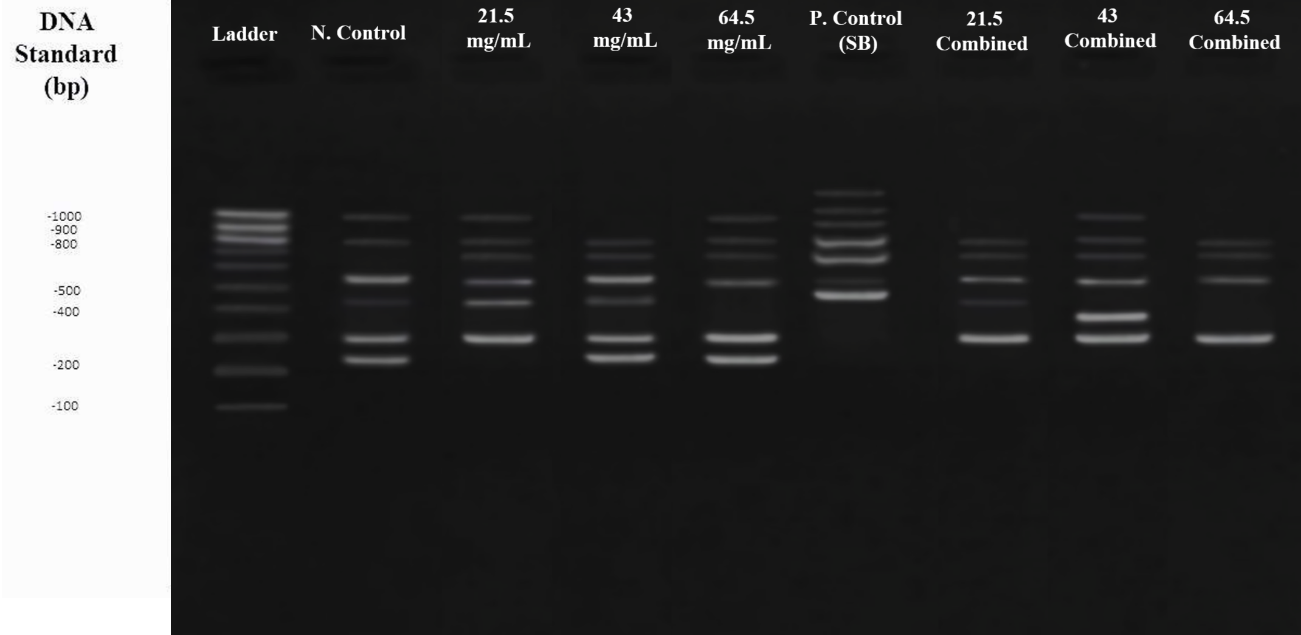


**Figure S6**. PCR products of ISSR-UBC-811 marker amplified with DNA treated with different concentrations of *S. oleraceus* extract as a single and combined treatment compared to negative control (dH₂O) for 48 hr.


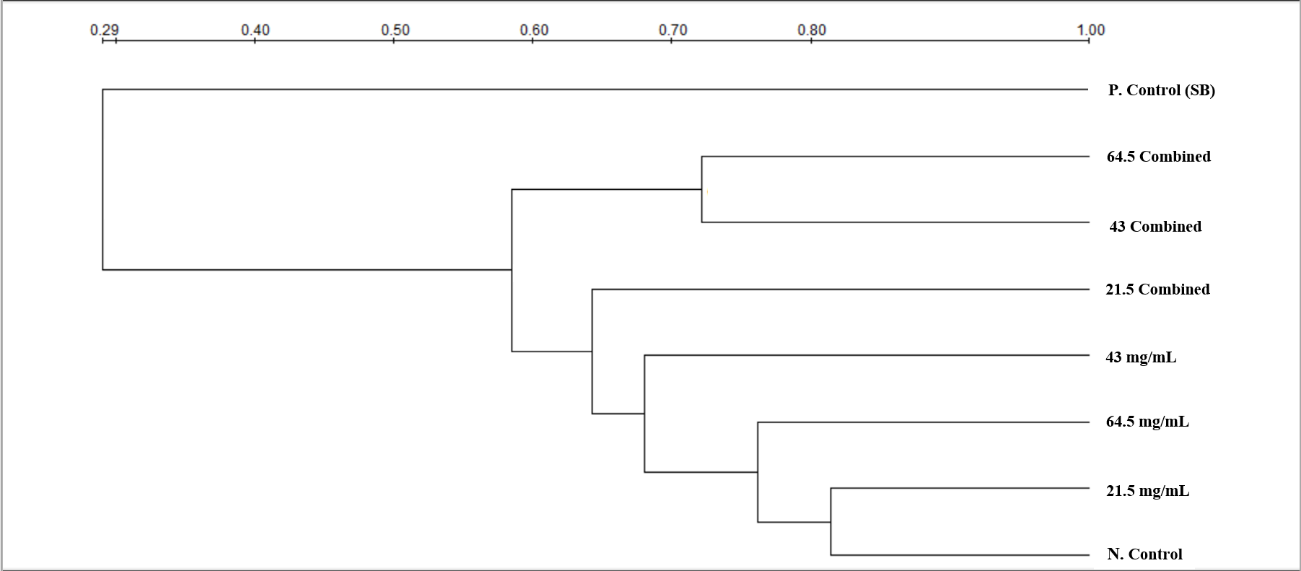


**Figure S7.** The phylogenetic by UPGMA of ISSR-UBC-811 marker for different concentrations and treatments of *S. oleraceus* extract as a single and combined treatment compared to the negative control (dH₂O).


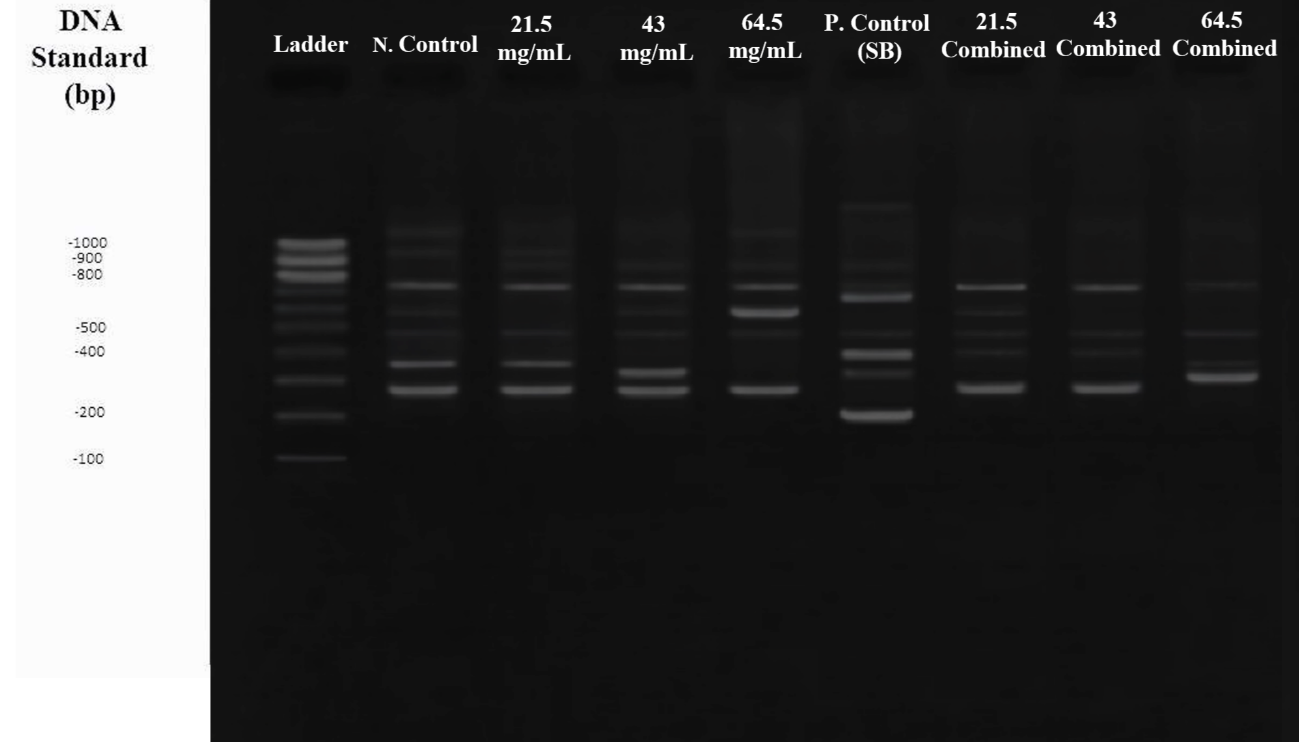


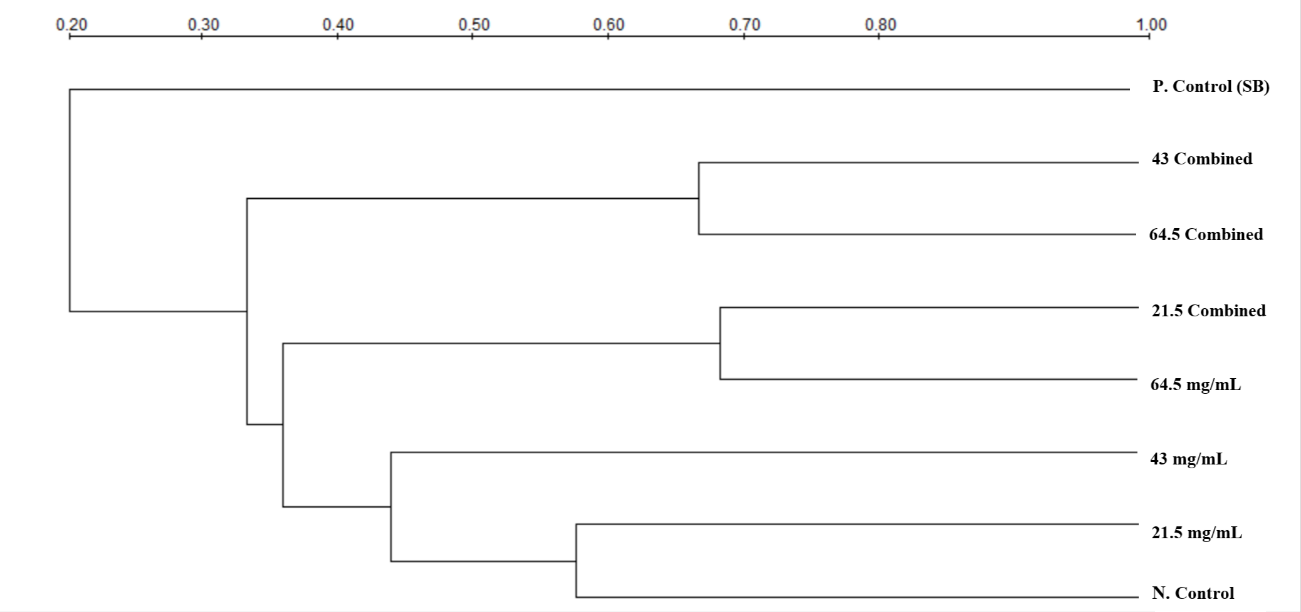
 **Figure S8**. PCR products of ISSR-MAO marker amplified with DNA treated with different concentrations of *S. oleraceus* extract as a single and combined treatment compared to negative control (dH₂O) for 48 hr.

**Figure S9.** The phylogenetic by UPGMA of ISSR-MAO marker for different concentrations and treatments of *S. oleraceus* extract as a single and combined treatment compared to the negative control (dH₂O).
